# Multiple decrement life tables of *Cephus cinctus* Norton (Hymenoptera: Cephidae) across a set of barley cultivars: The importance of plant defense versus cannibalism

**DOI:** 10.1371/journal.pone.0238527

**Published:** 2020-09-11

**Authors:** Buddhi B. Achhami, Robert K. D. Peterson, Jamie D. Sherman, Gadi V. P. Reddy, David K. Weaver

**Affiliations:** 1 Department of Land Resources and Environmental Sciences, Montana State University, Bozeman, MT, United States of America; 2 Department of Plant Sciences and Plant Pathology, Montana State University, Bozeman, MT, United States of America; 3 USDA ARS-Southern Insect Management Research Unit, Stoneville, MS, United States of America; University of Idaho, UNITED STATES

## Abstract

Accurately estimating cause-specific mortality for immature insect herbivores is usually difficult. The insects are exposed to abiotic and biotic mortality factors, causing cadavers to simply disappear before cause of mortality can be recorded. Also, insect herbivores are often highly mobile on hosts, making it difficult to follow patterns for individuals through time. In contrast, the wheat stem sawfly, *Cephus cinctus* Norton, spends its entire egg, larval, and pupal period inside a host stem. Therefore, with periodic sampling stage-specific causes of mortality can be ascertained. Consequently, we examined *C*. *cinctus* mortality in eight barley, *Hordeum vulgare* L., cultivars in two locations in Montana from 2016 to 2018 by collecting stem samples from stem elongation to crop maturity at weekly intervals, and collecting overwintered barley stubs the following spring and summer from the same plots. If larvae were present, we examined larval status—dead or alive—and categorized dead individuals into one of 5 mortality categories: plant defense, cannibalism, parasitism, pathogens, and unknown factors. We used multiple decrement life tables to estimate cause-specific mortality and irreplaceable mortality (the proportion of mortality from a given cause that cannot be replaced by other causes of mortality). Plant defense (antibiosis) caused 85.7 ± 3.6%, cannibalism (governed by antixenosis) caused 70.1 ± 7.6%, parasitism caused 13.8 ± 5.9%, unknown factors caused 38.5 ± 7.6%, and pathogens caused 14.7 ± 8.5% mortality in the presence of all causes of mortality. Similarly, irreplaceable mortality due to plant defense was 22.3 ± 6.4%, cannibalism was 29.1± 4.2%, unknown factors was 6.2 ± 1.8%, pathogens was 0.9 ± 0.5%, and parasitism was 1. 5 ± 0. 6%. Antibiosis traits primarily killed newly emerged larvae, while other traits supported more favorable oviposition decisions by females, increasing mortality by obligate cannibalism. Our results suggest that breeding barley for resistance to *C*. *cinctus* targeting both categories of traits (antibiosis and antixenosis) is a highly valuable tactic for management of this important pest.

## Introduction

Wheat stem sawfly, *Cephus cinctus* Norton (Hymenoptera: Cephidae), successfully adapted from its ancestral wild grass hosts to domesticated spring and winter wheat cultivars [1]. In addition, this species is not only widening its range southward to Colorado and Kansas [2], but it also is increasing infestation and larval survival in barley in Montana [3,4]. To mitigate economic loss, an integrated pest management (IPM) approach has been recommended for wheat across the northern Great Plains [5]. However, to further formulate and implement IPM tools against this pest, it is important to estimate the mortality of *C*. *cinctus* across life stages with other crop species in multiple environments.

*Cephus cinctus* is a univoltine insect. In spring, when plant hosts are at the stem-elongation stage, females lay eggs in suitable host stems. Approximately one week after oviposition, a larva emerges. The larva feeds primarily on the parenchymous tissues of the stem until the host plant reaches maturity. Once the host plant ripens and desiccates, the larva moves to the base of the stem, makes a v-shaped groove by chewing a uniform gouge around the stem interior, and plugs the stem below this groove with frass and plant materials to make a hibernaculum. The v-shaped groove contributes to stem lodging when gravity and wind impact the ripened stem. The remaining part of the lodged stem that is intact in the soil and contains an overwintering larva is called a “stub.” Within this stub, the larva moves close to the root crown where it hibernates and spends 8−9 months as a prepupa. The following spring, and after sufficient warming, the hibernated prepupa fully metamorphoses before chewing through the frass plug in the stub and emerging as an adult [6,7].

The probability of death of an individual at each developmental stage due to cause-specific mortality can be estimated using a multiple decrement life table [8]. The multiple decrement life table mathematically accounts for a number of mutually exclusive causes of mortality. In addition, the multiple decrement life table can be used to estimate the contribution of each cause of mortality in the presence and absence of other mortality causes.

Parasitism, host plant resistance, and cannibalism are three major causes of mortality of *C*. *cinctus* in wheat [9]. However, the mortality due to parasitism varies by locations and by host types [10,11]. These three major causes of mortality act on different stages of *C*. *cinctus* larvae. For instance, host plant defense kills mostly neonates, at an early stage of development, while cannibalism and parasitism occur in instars larger and later than neonates. The larvae that survive plant defense mechanisms are still vulnerable to parasitism and are more vulnerable to cannibalism if multiple live larvae inhabit in a stem [12–14]. Thus, cannibalism and plant defense are important factors influencing *C*. *cinctus* fitness at both the individual and population levels. However, the role and importance of these three causes of mortality—plant defense, cannibalism, and parasitism—for *C*. *cinctus* developing in a barley (*Hordeum vulgare L*.) hosts is yet to be determined.

Although barley was historically categorized as resistant to *C*. *cinctus*, a decade-long study revealed an increasing infestation rate and larval survival rate of *C*. *cinctus* in barley in Montana [4]. Montana ranked second among barley producing states based on the amount of production in the US in 2019 [15].

Growing solid-stem—filled with pith that reduces the larval survival rate [16–18]—cultivars and swathing are two tools that can mitigate economic loss incurred by *C*. *cinctus* in Montana wheat crops [19]. However, both management tactics are not implementable for barley because all currently cultivated barley cultivars have hollow stems and swathing can substantially reduce grain quality, especially in malt barley. In Montana, malt barley was 61.8% of total barley production by hectare in 2019 [20].

To reduce the increasing economic losses caused by *C*. *cinctus* in Montana, estimating age- and cause-specific mortality in populations is crucial. Cannibalism and host plant resistance are two major causes of mortality in wheat [9]. Both causes of mortality are mediated by host plant traits. For instance, cannibalism ultimately occurs because the host plants release attractive compounds like (*Z*)-3-hexenyl acetate [21,22] and (*E*)-and (*Z*)-β-ocimene to gravid females [23]. These, along with other physical characters, stimulate the abundant gravid females to lay numerous eggs in a stem at high population densities [22,23]. In wheat, stem solidness obstructs larval movement and most of the larvae eventually die [18,24]. The pith also plays a negative role in maternal oviposition decisions in stems with varying solidness due to physically encountered factors [25] and because the hollow stem wheat cultivar releases greater amount of (*Z*)-3-hexenyl acetate compared to solid stem wheat [26]. Therefore, estimating causes of mortality by barley cultivars will provide a foundation to estimate cultivar-specific mortality and may allow for the development of cultivars that cause consistently greater levels of larval mortality. Consequently, in this study, we estimated age- and cause-specific mortality of *C*. *cinctus* in eight barley cultivars (Celebration, Champion, Craft, Haxby, Haybet, Hockett, Lavina, and Tradition) at two locations in Montana.

## Materials and methods

### Field site, field preparation, and seeding

To estimate *C*. *cinctus* mortality in barley, we conducted a field study in Montana near Amsterdam in 2016 (45°45’27.3” N, 111°24’00.9” W) and 2017 (45°45’33.2” N, 111°23’50.0” W), and near Big Sandy in 2017 (48°15’42.1” N, 110°22’19.1” W). In each site-year, we planted eight barley cultivars in the spring. The cultivars were ‘Haxby’ and ‘Champion’ (feed barley); ‘Haybet’ and ‘Lavina’ (forage or hay barley); and ‘Celebration,’ ‘Craft,’ ‘Hockett,’ and ‘Tradition’ (malt barley). All cultivars were two-rowed seeded heads except ‘Celebration and ‘Tradition’, which had six-rowed heads.

Each study used a randomized complete block design. The study field was divided into three blocks based on field variability due to slope. Each block was divided into eight plots and each plot was 1.8 m × 3.6 m with a 0.3-m spacing between each plot. The seeding rate was 9 g/m^2^. We performed several manual weeding operations to maintain a better crop stand.

### Summer samples

We began stem sampling 59 days after seeding because more than 50% of plants were at stem elongation [27]. The stem elongation stage of the host plant is when *C*. *cinctus* females can first lay eggs because they do not deposit eggs on foliage–or on the later developing seed heads, for that matter. The sampling continued at weekly interval to crop maturity; in total, we collected for 9 consecutive weeks. The procedure for stem sampling was conducted as described previously [3]. For each sampling week, we randomly measured a 0.3-m row length in each plot, uprooted all the plants within the measured length, and wrapped the uprooted plants in a uniquely labelled paper bag. We repeated this uprooting procedure 3 times within a plot; so that each plot had 3 bags of samples per sampling week. Subsequently, we dissected the collected samples lengthwise and, if infested, recorded the status of *C*. *cinctus* by counting eggs and dead or live larvae. We selected only 35 primary stems from each bag of the samples (the sample that we collected until the 8^th^ week) because all samples could not be dissected on the day they were collected. Unprocessed samples were stored in a 4°C storage room and we dissected them as rapidly as possible on the following day to prevent wilting and to maintain larval status as when collected. In total, we dissected 105 stems per plot per sampling week. However, we dissected all ripened stems from the harvest samples collected at crop maturity (9^th^ week of sampling).

### Spring and summer stub samples

We collected stub samples from each plot where we collected the stem samples during the previous summer. We collected stubs twice, at both before and after the flight period of adults. Once again, these stubs are the remaining lower part of a cut stem where the hibernating larva is located.

The first collection of stubs was conducted in the first week of the following April or May (Amsterdam 2016: 4 April 2017; Amsterdam 2017: 28 April 2018; Big Sandy 2017: 3 May 2018). In mid to late May through early July, female adults emerge, mate, and begin laying eggs. Therefore, we named these collected samples “pre-emergence” or “pre-flight period.” For stub collection, we divided each plot into two equal sections. From the first half of each remaining plot area, we uprooted all the stubs and wrapped these inside a uniquely labelled paper bag. Thereafter, we randomly selected at least 25 stubs that contained hibernaculae with diapaused larvae from each plot and recorded the status, dead or alive, of the overwintered larvae after dissection. We selected 25 stubs to maintain uniform sample size across all cultivars and sites. Additionally, we categorized the dead larvae by mortality causes, including parasitism, pathogens, or unknown factors.

Next, in July (Amsterdam 2016: 10 July 2017; Amsterdam 2017: 12 July 2018; Big Sandy 2017: 20 July 2018), we collected the remaining stubs from each plot which we considered “post-emergence” samples. Then, we again randomly separated 25 stubs per plot, dissected them and recorded the status as either emerged or dead. For assessing potential emergence, we additionally considered whether the top part of the stub, the frass plug, had only a single emergence hole and did not have any indicators of larval or pupal mortality, such as a cadaver or more correctly, fragments of a cadaver, or a parasitoid cocoon. Otherwise, we recorded the life stage as dead by the appropriate cause of mortality.

### Causes of mortality

As described, we collected samples for 3 stages of *C*. *cinctus* development that occur within a stem: summer samples (pre-diapause period), overwintered (pre-flight period), and post emergence (at the end of the flight period). After determining the status, we categorized the death of an individual by one of the following mortality causes: cannibalism, plant defense, parasitism, pathogens, or unknown factors.

Within obligate cannibalism events, we categorized two groups. The first was egg cannibalism when we found a stem with multiple eggs or a larva and an egg(s). The second was larval cannibalism if a stem contained more than one larva. In both cases, a single larva eventually consumes all others, both eggs and larvae, in that stem. Only a single larva per infested stem proceeds to cut the stem and hibernate if the larva survives other causes of mortality [14,24,28,29].

We categorized plant defense as a cause of death if we found a stem contains dead neonates or the stem lining had only couple of millimeters of feeding scars at the larval emergence site as recently described by Buteler et al [9]. Additionally, if we saw only feeding scars, tiny amounts of frass or a small fragment of a dead neonate in the stem, then we also categorized the cause of death as plant defense.

We combined the mortality due to parasitoids and predators under the category of parasitism because we only found two stems with larvae of the clerid predator, *Phyllobaenus dubius* Wolcott (Coleoptera: Cleridae), described in Morrill et al. [30], at the site near Amsterdam in 2016. Thus, otherwise for this paper, “parasitism” represents a stem containing either a parasitoid larva, a parasitoid cocoon or a diagnostic emergence hole in the stem wall. For the rate of parasitism, we categorized the larvae into two groups based on their relative size: early growth stages or stages vulnerable to parasitism. The early larval growth stages are not vulnerable to parasitism. Therefore, an early stage before parasitism was possible occurred from the 2^nd^ to 4^th^ week of sampling and the records for the period with stages vulnerable to parasitism occurred from the 5^th^ to 9^th^ week of sampling, as well as being evident in stub samples taken for the pre- and post-flight periods.

If the larval cadaver was covered with white or pink hyphae or was characteristically pink [31], we assumed the cause of death was entomopathogenic fungi and we categorized it as “pathogens.” If the larva mined through multiple internodes but was found dead from other than the above-mentioned mortality causes, it was classified as having died due to “unknown factors.”

### Construction of multiple decrement life tables

To construct multiple decrement life tables, we used abridged life tables to calculate mortality causes and relevant mortality proportions by those causes in the presence of other mortality causes [32]. We produced the multiple decrement life tables by cultivar and study site using the spreadsheet program M-DEC [33]. After accounting for mortality causes from summer samples, as well as pre-emergence and post emergence stubs, we calculated the percentage of mortality for each cause of mortality in the presence of other mortality causes, and subsequently the irreplaceable mortality. Irreplaceable mortality is mathematical estimates of the degree of impact of a specific mortality factor if it was removed from the mix of mortality factors operating on the insect at the time the study was conducted. For each specific cause, the irreplaceable mortality was determined using the methods described by [10,32–34]. In this technique, the variables are defined as: x = life stage index, l_x_ = the number of individuals alive at each x, d_x_ = the total number of deaths in each stage, al_x_ = the proportion of the cohort living at the beginning of the stage (starting at 1.0 for the first state and calculated by alx-1—ad_x-1_), ad_ix_ = proportion of death attributable to one cause, ad_x_ = proportions of deaths from all causes (ad_1x_ + ad_2x_ + … ad_5x_), aq_x_ = stage specific probability of dying from all listed causes (d_x_/l_x_).

Similarly, the probability for cause of death in the absence of other causes can be estimated using a quadratic solution. Elimination-of-cause analysis relies on the probability of surviving each source of mortality (*px*) and its complement (1 –*qx*) where (1 –*q*_*1*_) x … x (1 –*q*_*n*_) is the chance of jointly surviving a set of mortality causes and its complement, 1 –[(1 –*q*_*1*_) x…x (1 –*q*_*n*_)] is the chance of jointly dying from a set of mortality causes. To estimate mortality in the absence of one or more causes, two simultaneous equations with two unknowns are used. For example, by expression *q*_*1*_ (e.g. cannibalism) in terms of *q*_*2*_ (e.g. all other mortality causes), *D*_*1*_ and *D*_*2*_ (the proportion of all individuals observed that died of cause 1 and cause 2), this yields the quadratic equation:

$$aq_{2}{}^{2}+bq_{2}+c=0,\;\mathrm{where}\;a=D_{1},\;b=–\left(D_{1}+D_{2}\right),\;\mathrm{and}\;c=D_{2}\left(D_{1}+D_{2}\right).$$

The value of *q*_*2*_ can be calculated by substituting a, b, and c in the quadratic formula. Similarly, to calculate irreplaceable mortality for any cause of mortality, for instance irreplaceable mortality due to cannibalism, we subtracted total mortality caused by all estimated mortality causes (cannibalism, parasitism, plant defense, pathogens, and unknown) from the total mortality (parasitism, plant defense, pathogens, and unknown) except for the mortality from cannibalism [10,33].

### Data analyses

We compared the percentage mortality of each mortality cause in the presence of other mortality causes by cultivars and by sites. In addition, we compared percentage of irreplaceable mortality caused by each mortality cause by cultivars and by sites. We used linear and nonlinear mixed effects model (nlme) [35] with percentage mortality attributed to each cause as the response variable, while cultivars, sites and their interaction were fixed effects. Year was considered as a random factor for the analysis. All the percentage data were square-root transformed to meet the assumption of homogeneity of variance, but untransformed means and the associated standard errors are presented. First, we included a site x cultivar interaction for the analysis and later dropped it if *P* > 0.05 for interaction. Post-hoc analysis was done whenever *P* ≤ 0.05 by using Tukey HSD (multcomp) [36]. Tukey HSD was used to reduce error due to unequal sample sizes across cultivars. We used the package ‘scales’ [37] to prepare a heat map to visualize mortality percentage by mortality causes, by cultivars, and by site × year for the *C*. *cinctus* stages on visualized life tables. Additionally, using the packages ggpubr [38] and ggcorrplot [39], we prepared correlation plots, by cultivars, to show relationship between mortality percentage caused by plant defense and cannibalism. All data were analyzed in Comprehensive R Archive Network (CRAN) version 3.5.3 [40] and visualization by using ggplot2 [41].

## Results

**Fig 1:**
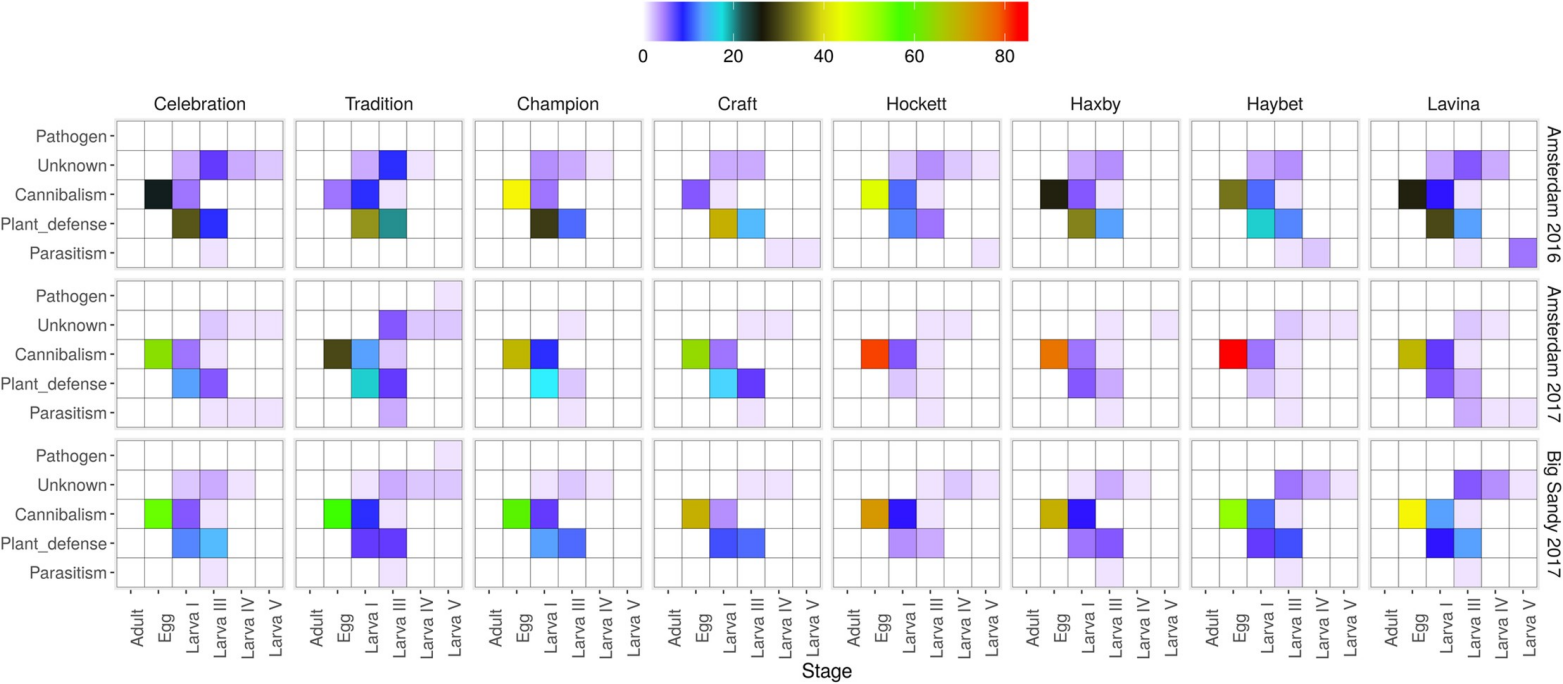
Heat map displaying the percentage of mortality by each cause of mortality at each developmental stage for *Cephus cinctus* in barley cultivars that were grown in Montana. Larva I: Pre-parasitism period; Larva III: Parasitism period; Larva IV: Overwintered larva (pre-flight period; post-parasitism); Larva V: Overwintered larva (post-flight period; post-parasitism).

A total of 64,076 stems and 3,200 stubs were split to assess *C*. *cinctus* mortality among eight cultivars over 3 site × years. Multiple decrement life tables were produced for each study site and each cultivar (S2 Table and Fig 1). Cannibalism, plant defense, and unknown factors were the three main causes of mortality for eggs through pre-diapause larvae, whereas unknown factors, pathogens, and parasitism were main causes of mortality recorded for large larvae before and after overwintering (Table 1, Fig 1, S1 and S2 Tables).

**Table 1:** Multiple decrement life tables of wheat stem sawfly, *Cephus cinctus*, in barley.

Category x	Alive l_x_	Proportion of dying aq_x_	Proportion of living al_x_	Proportion of total dying ad_x_	Parasitism aq_1x_	Plant defense aq_2x_	Cannibalism aq_3x_	Unknown aq_4x_	Pathogens aq_5x_
**Egg**	8968	0.619	1	0.619	0	0	0.619	0	0
**Larva I**	18447	0.478	0.381	0.182	0	0.102	0.072	0.008	0
**Larva III**	17254	0.554	0.199	0.110	0.010	0.063	0.009	0.027	0
**Larva IV**	1546	0.169	0.089	0.015	0.002	0	0	0.012	0.0007
**Larva V**	1499	0.1368	0.074	0.010	0.002	0	0	0.006	0.0013
**Adult**	1291	0	0.064	0.064					
**Total**				1	0.015	0.165	0.701	0.054	0.002

Larva I: Pre-parasitism period; Larva III: Parasitism period; Larva IV: Overwintered larva (pre-flight period; post-parasitism); Larva V: Overwintered larva (post-flight period; post parasitism).

aq_x_ = proportion of death caused by all the given mortality causes in stage x given that the individual is alive at the beginning of stage x; al_x_ = proportion of survivors at stage x out of original cohort of all individuals; ad_x_ = proportion of deaths in stage x from all the mortality causes; aq_ix_ = proportion of death from cause i in stage x in the presence of all other mortality causes given that the individual is live at the beginning of state x.

The probability of death from a specified cause in the presence of combinations of other causes by all stages is shown in S1 Table. For instance, cannibalism alone would kill 71% of population (S1 Table). The combination of three mortality causes—cannibalism, unknown factors, and plant defense—would kill 92% of the population, while the combination of all 5 listed causes of mortality would kill 93% of the population. Thus, the effect of mortality due to parasitism or pathogens for the population in the presence of the other 3 causes was negligible (Table 1, S1 Table).

**Fig 2:**
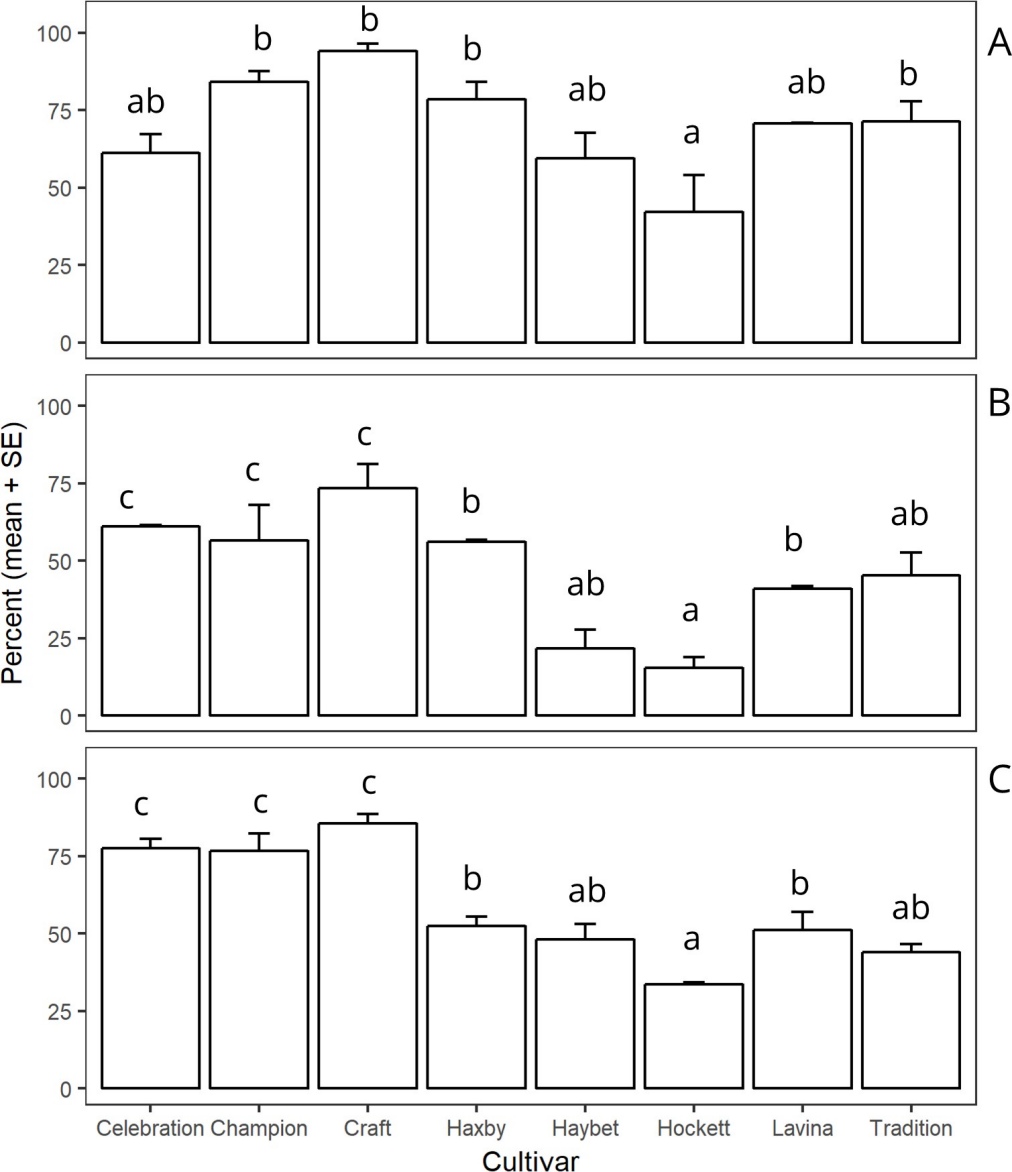
Mean mortality percentages due to plant defense by site and cultivar. The percentage of mortality was calculated in the presence of other causes of mortality. Bars within the site followed by different letters are significantly different (α = 0.05). Panel A: Amsterdam 2016 (F _7, 14_ = 6.25, *P* = 0.001); Panel B: Amsterdam 2017 (F _7,7_ = 14.80, *P* = 0.001); Panel C: Big Sandy 2017 (F _7, 14_ = 25.05, *P* < 0.001). SE is standard error of the mean.

There was an interaction between site × years and cultivars for the percentage of larval mortality caused by plant defense (F _14,35_ = 3.09, *P* = 0.003). However, the cultivar ‘Hockett’ had the lowest larval mortality due to plant defense compared to the other cultivars at all sites (Fig 2).

**Fig 3:**
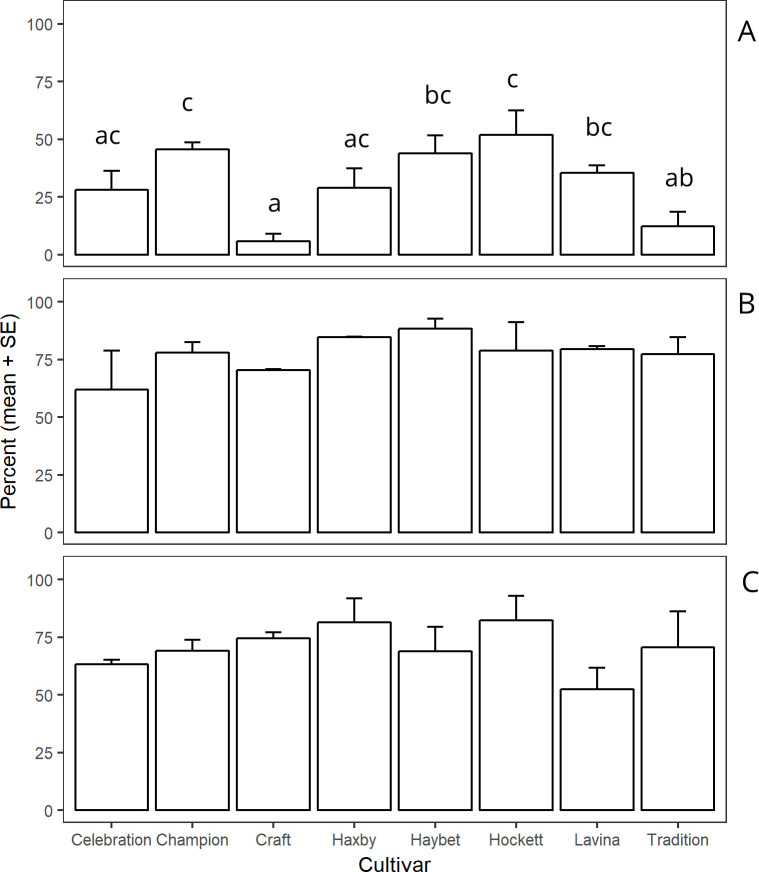
Mean mortality percentages due to cannibalism by site and cultivar. The percentage of mortality was calculated in the presence of other causes of mortality. Bars within the site followed by different letters are significantly different (α = 0.05) and the bars without letters within the site are not different (α = 0.05). Panel A: Amsterdam 2016 (F _7, 14_ = 6.11, *P* = 0.002); Panel B: Amsterdam 2017 (F _7, 7_ = 0.95, *P* = 0.523); and Panel C: Big Sandy 2017 (F _7, 14_ = 1.008, *P* = 0.465). SE is standard error of the mean.

Mortality from cannibalism was significantly different for site × cultivar (F _14, 35_ = 4.12, *P* = 0.004). The variation in mortality due to cannibalism was greatest near Amsterdam in 2016 when compared with near Amsterdam in 2017 and near Big Sandy in 2017 (Fig 3).

**Fig 4:**
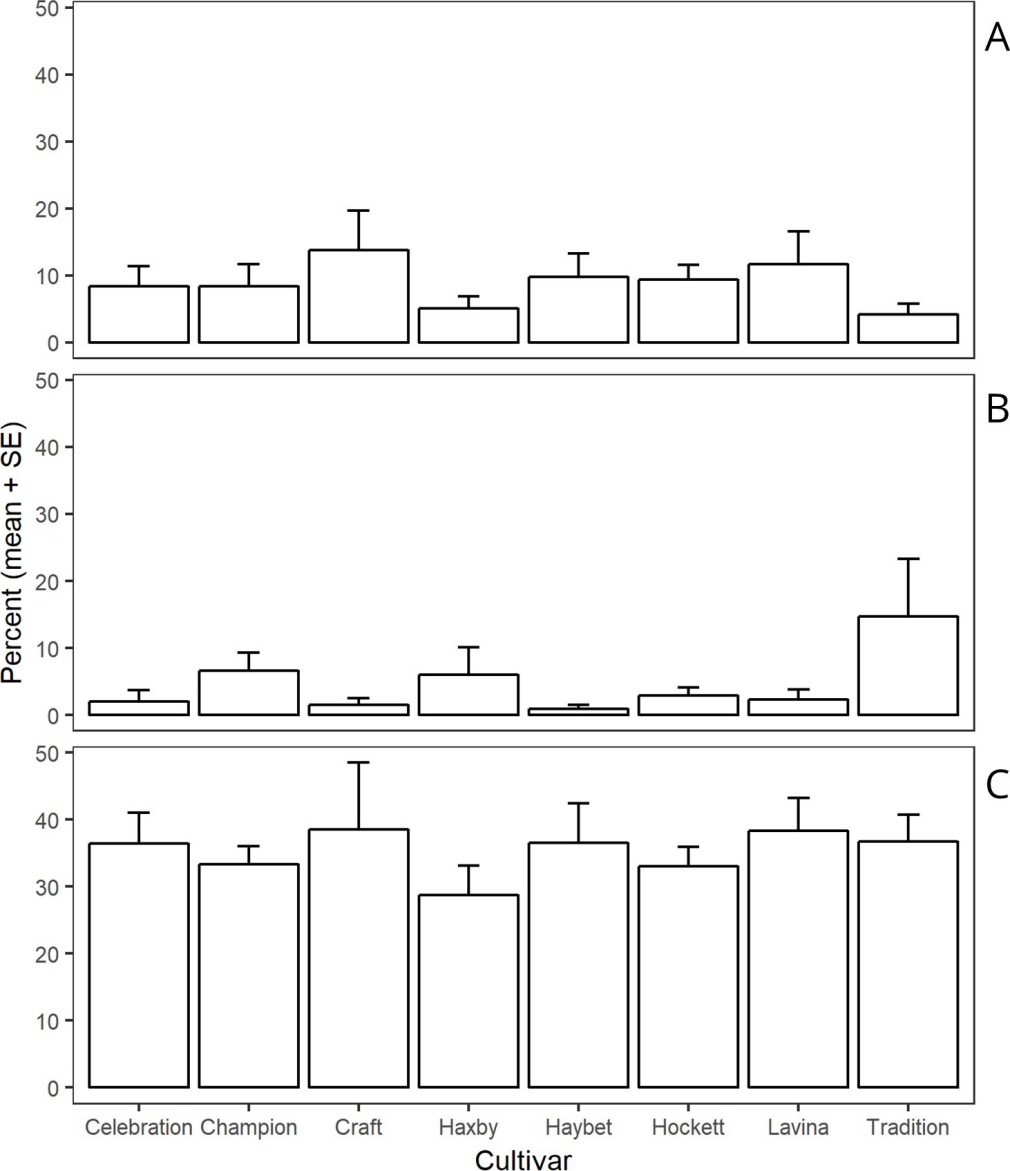
Mean mortality percentages by parasitism, pathogens, and unknown factors by site and cultivar. The percentage of mortality was calculated in the presence of other causes of mortality. Bars without letter within the mortality causes are not different (α = 0.05). Panel A: Parasitism (Cultivar: F _7, 35_ = 1.40, *P* = 0.233); Panel B: Pathogens (Cultivar: F _7, 35_ = 1.47, *P* = 0.208); Panel C: Unknown factors (Cultivar: F _7, 35_ = 1.31, *P* = 0.248). SE is standard error of the mean.

Parasitism and pathogens caused less than 20% mortality individually, while unknown factors caused approximately 30% (Fig 4). All sites had a similar pattern of mortality within each cause of mortality in all cultivars (Parasitism: F _14, 35_ = 1.833, *P* = 0.073; Pathogens: F _14, 35_ = 0.637, *P* = 0.815; Unknown factors: F _14, 35_ = 1.314, *P* = 0.248).

There was no site × year effect on irreplaceable mortality by cultivars within the specified cause of mortality: cannibalism (F _14, 35_ = 0.97, *P* = 0.493), pathogens (F _14, 35_ = 0.97, *P* = 0.494), plant defense (F _14, 35_ = 0.74, *P* = 0.715), and unknown factors (F _14, 35_ = 0.96, *P* = 0.502). The irreplaceable mortality due to cannibalism in ‘Hockett’ was greater than for the other cultivars (Table 2). Similarly, irreplaceable mortality due to pathogens was the lowest compared to other causes of mortality (Table 2).

**Table 2:** Irreplaceable mortality by cause of mortality and cultivar.

Cultivar	Irreplaceable mortality percent (mean ± SE*)				
	Plant defense	Cannibalism	Unknown factors	Pathogens	Parasitism
**Celebration**	18.74 ± 2.69	8.96 ± 1.78	5.81 ± 1.79	0.11 ± 0.07	1.03 ± 0.47
**Champion**	17.02 ± 3.33	9.41 ± 2.17	2.24 ± 0.25	0.45 ± 0.23	0.40 ± 0.16
**Craft**	21.65 ± 7.05	5.59 ± 1.92	1.72 ± 0.46	0.04 ± 0.03	0.26 ± 0.12
**Haxby**	17.27 ± 6.08	16.10 ± 3.87	2.75 ± 0.96	0.51 ± 0.37	0.27 ± 0.08
**Haybet**	12.26 ± 4.35	18.38 ± 2.56	5.32 ± 1.40	0.10 ± 0.07	0.77 ± 0.28
**Hockett**	6.42 ± 2.60	29.15 ± 4.19	4.92 ± 1.06	0.40 ± 0.19	1.41 ± 0.41
**Lavina**	15.61 ± 3.72	12.80 ± 2.96	6.44 ± 1.22	0.28 ± 0.18	1.10 ± 0.39
**Tradition**	22.29 ± 6.35	11.13 ± 3.82	6.22 ± 1.80	0.39 ± 0.25	1.03 ± 0.10
**DF**	7,49	7,49	7,49	7,49	7,49
**F-value**	2.44	11.35	2.69	1.16	4.77
***P*-value**	0.037	<0.001	0.024	0.347	<0.001

*SE is standard error of the mean.

**Fig 5:**
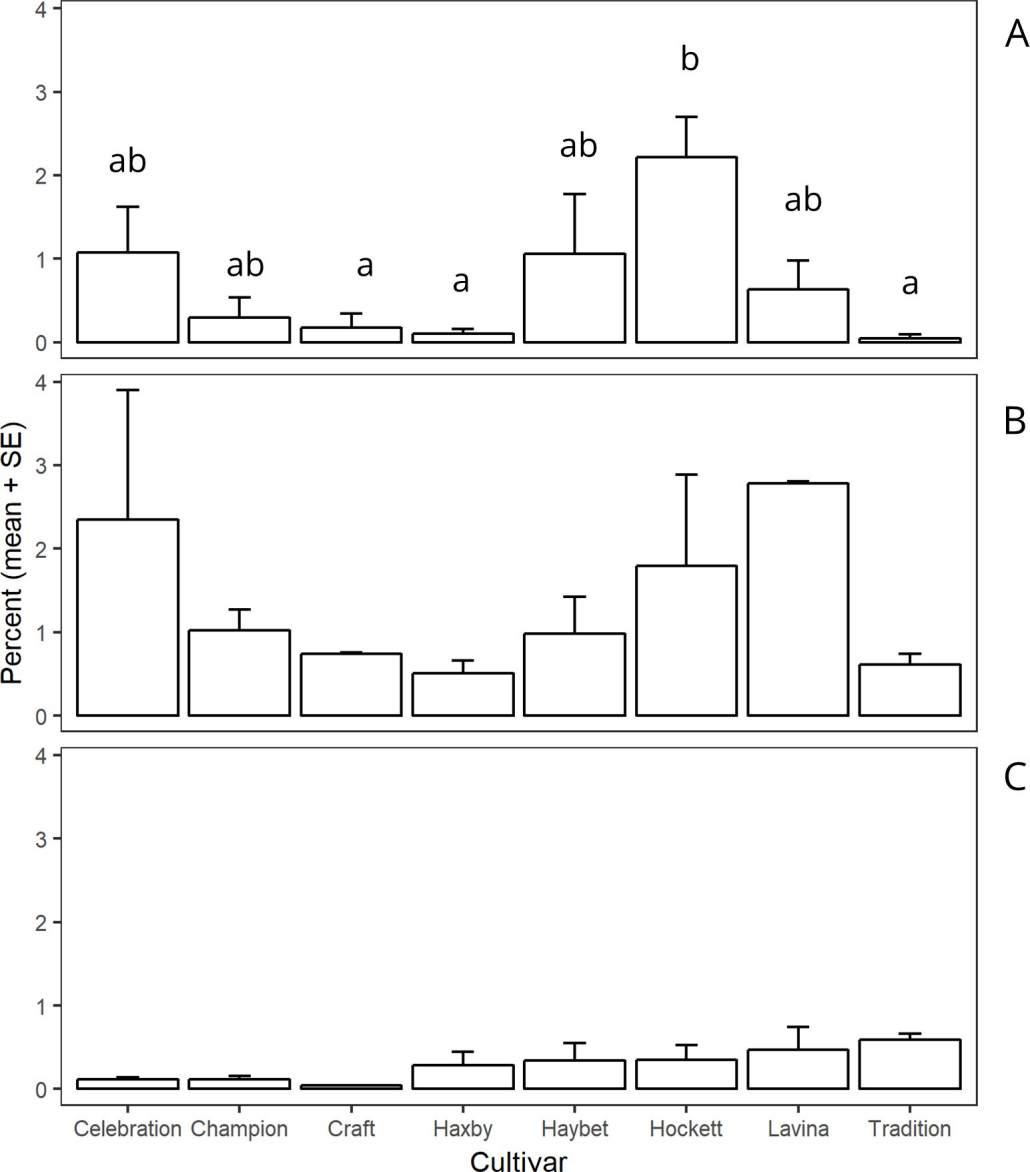
Mean percentages of irreplaceable mortality due to parasitism by site and cultivar. Bars within the site followed by different letters are significantly different (α = 0.05) and the bars without letters within the site are not different (α = 0.05). Panel A: Amsterdam 2016 (F _7,14_ = 3.96, *P* = 0.013); Panel B: Amsterdam 2017 (F _7,7_ = 2.30, *P* = 0.146); Panel C: Big Sandy 2017 (F _7,14_ = 1.86, *P* = 0.152). SE is standard error of the mean.

Irreplaceable mortality due to parasitism was less than 4% but was significantly different by cultivar and by site × year (F _14, 35_ = 2.19, *P* = 0.029) (Fig 5). The lowest irreplaceable mortality due to parasitism was found near Big Sandy in 2017.

## Discussion

Our findings indicate that plant defense, cannibalism, and unknown factors were the three major causes of mortality for *C*. *cinctus* developing in barley (Table 1). Although irreplaceable mortality due to plant defense and cannibalism was similar to previous reports for wheat [9,10], the mortality because of parasitism and pathogens was quite low in our study compared with previous observations made in wheat. Both cannibalism and plant defense are mediated by host plant traits, either relative preference by gravid females [26] or antibiosis that limits larval survival rate. Thus, capitalizing on cultivar traits that cause both a greater proportion of antibiotic neonate mortality and those traits that result in obligate cannibalism when attracting more females to oviposit [23,42] are critical considerations to reduce economic losses caused by this species.

Our results indicate that cannibalism caused the greatest mortality in the presence of other mortality causes (Fig 3) and high levels of irreplaceable mortality (Table 2). The mean irreplaceable mortality due to cannibalism was 5.59 ± 1.29% in ‘Craft’ and 29.15 ± 4.19% in ‘Hockett’ (Table 2). Buteler et al. [9] observed similar variation in irreplaceable mortality due to cannibalism when comparing susceptible (hollow stem) and resistant (solid stem) wheat cultivars.

Cannibalism is obligate in *C*. *cinctus* when a stem contains multiple eggs [9,24,28]. Why a cohort of *C*. *cinctus* females deposit multiple eggs in a stem over a flight period of several weeks is not known, but the following could be potential reasons. First, females prefer to lay eggs in hosts that release greater amounts of attractive volatiles such as *β*-ocimene, (*Z*)-3-hexenyl acetate, and (*Z*)-3-hexenol acetate [23,26] and therefore may indiscriminately lay eggs in a host even though conspecific females have already laid eggs [22,43]. Second, the females are short-lived, typically surviving about 7 days, which may provide an overarching imperative to lay eggs in any available host instead of dying with a greater complement of eggs while searching for an optimal host. For instance, in the absence of primary hosts for oviposition, *C*. *cinctus* can lay eggs in flax stems [44] and in wheat straw [45]. The behavior of accepting a lower quality host for oviposition in the absence of higher quality hosts can be accompanied by higher levels of cannibalism on less suitable hosts in phytophagous insect species [46]. However, in our study more cannibalism occurred in cultivars with less evidence of plant defense which also received more eggs; these cultivars are arguably more, rather than less suitable as hosts. Regardless of which of these reasons cause females to lay multiple eggs in a stem, our results strongly suggest cannibalism plays a considerable role in influencing the population dynamics of *C*. *cinctus* in barley compared to other mortality causes.

Multiple eggs in a stem are not laid by a single female [22,25], but rather by more than one female because of failure to recognize a previously infested stem [22]. This non-discriminatory behavior suggests neither self-provisioning nor opportunistic oviposition facilitates the occurrence of cannibalism in larval *C*. *cinctus*, as might be expected from studies on species that oviposit in clutches, some of which may have evolved parental guarding [47–49]. For example, ‘Craft,’ ‘Champion,’ and ‘Celebration’ had fewer stems with multiple eggs so that they had a lower percentage of mortality due to cannibalism compared to ‘Hockett.’ Fewer eggs per stem were likely because these cultivars were less attractive to foraging gravid females. This host-mediated trait of fewer eggs in a stem is somewhat similar to those observed for solid-stem wheat [25], which inhibits the deposition of eggs when probing with the ovipositor.

Individual females do not sacrifice their own eggs by laying multiple eggs in a stem to avoid cannibalism. Cannibalism poses risks to survivors of being infected by species-specific pathogens from their kin and potential mortality due to fighting [50]. We hypothesize that having multiple eggs from different females in a stem is mediated primarily by host plant volatiles and not by cues released from already oviposited eggs [22], yet this strategy favors increased cannibalism.

Cannibalism is a major cause of mortality. From the perspective of population ecology, cannibalism seems to be an important cause of mortality when considering recruitment and it possibly has fitness implications in the host that contains defense compounds or lacks essential nutrients [51]. Thus, fitness can potentially be improved by gaining additional nutritional resources or by reducing the rate of parasitism to larvae [14,52].

Further, cannibalism in other species is a well-known phenomenon to gain additional nutritional resources. For instance, *Harmonia axyridis* Pallas shortens development time and increases survivorship by consuming conspecific larvae than by consuming prey aphid species that sequester plant compounds as a defense mechanism [53]. By consuming conspecific larvae, *H*. *axyridis* avoids the potential negative effect of plant toxins [53]. Several previous reports [4,54] and the current study have indicated that a large proportion of *C*. *cinctus* neonate mortality indicates that either the host stem tissues contain antibiotic toxins, or possibly the tissues lack some essential nutrients required for neonate growth and development (categorized as plant defense: Table 2, Fig 2). But by consuming conspecific larvae and eggs, first, each cannibal avoids both tissue toxins or diet deficiency and acquires additional nutrient resources. Second, due to extra nutritional resources, cannibalizing individuals would be more vigorous and could also consume vulnerable parasitized hosts, effectively eliminating the larval ectoparasitoids [13,14]. Third, cannibalism reduces larval host density available for parasitism and can ultimately reduce the rate of parasitism [52,55]. Cannibalism also reduces injury to stem tissue by eliminating other feeding individuals, thus limiting tritrophic communication to parasitoids that could immediately favor the infested stem [56]. Thus, cannibalism can increase the fitness of survivors and populations in this system [57].

Our results show that mortality due to plant defense caused the second-highest percentage of mortality (Table 1, S1 Table), while irreplaceable mortality due to plant defense was greatest (Table 2). For example, in the cultivar ‘Craft,’ the percentage of irreplaceable mortality due to plant defense was 4-fold higher than the irreplaceable mortality due to cannibalism, 12-fold higher than irreplaceable mortality due to unknown factors, and almost 20-fold higher than irreplaceable mortality due to parasitism or pathogens (Table 2 and Fig 5). Thus, ‘Craft’ has stronger antibiosis traits compared to the other cultivars.

In wheat, a higher proportion of larval mortality occurs in pith-filled solid stems than in hollow stems [18,58]. Even though all barley cultivars in this study have hollow stems, our results reveal the percentage of mortality due to plant defense was higher than the mortality reported in any solid-stem wheat cultivar. Therefore, we suggest that larval mortality in barley is likely caused by traits other than those associated with stem architecture.

Barley plants synthesize the defensive alkaloids hordenine (N, N-dimethyltriamine) and gramine (N, N-dimethylindolemethyl-amine), which are feeding deterrents to *Heliothis* spp. [59] and aphids [60,61]. Further, barley plants synthesize protease inhibitors in the form of trypsin inhibitors [62] that decrease the access to essential amino acids and impair protein functions that eventually increase the mortality rate in the spider mite, *Tetranychus urticae* Koch (Arachnida: Tetranychidae) [63]. However, it was beyond the scope of our study to identify associated physical strength measurements or biochemical mechanisms for stem tissue, which can collectively be considered antibiosis, although these both may play important roles in resistance to *C*. *cinctus*.

Although both cannibalism and plant defense were mortality causes mediated through plant traits, they were negatively correlated (S1–S4 Figs). Plant defense kills neonates at the site of emergence, and it is density independent. Cannibalism occurs after larvae gain a certain size and it is density dependent. Thus, cannibalism allows for survival of one individual in a cohort of multiple immatures within a single stem. As a result, the surviving mature larvae cut the stems in preparation for overwintering, which is agronomically undesirable [19]. Further, due to cannibalism, cannibals consume less stem tissue, which could be a bridge for this species when adapting from wild grass to domesticated wheat and currently, to barley. However, both causes of mortality need to be considered to best manipulate cultivars traits against *C*. *cinctus*.

Irreplaceable mortality due to pathogens was lowest across all cultivars at < 1% (Table 2). Similarly, Buteler et al. [9] observed that pathogens contributed a small fraction (< 5%) to the mortality of *C*. *cinctus* in wheat. We did not assess which pathogen species killed larvae. Studies show that entomopathogenic fungi [64,65] and certain *Fusarium* spp. [31] can colonize plant tissue without developing highly apparent symptoms in the plants, but these fungi can kill diapausing larvae [31,65]. Thus, our results suggest that barley cultivars may also harbor entomopathogenic fungi, but the level of mortality was negligible.

The overall parasitism rate was < 20% in the presence of other causes of mortality across all cultivars and sites (Table 1). Similarly, irreplaceable mortality due to parasitism was < 4% (Fig 5, Table 2). Conversely, in wheat, several other studies have reported that *C*. *cinctus* mortality due to parasitism was as much as 60% [9,10,54,66] and irreplaceable mortality due to parsitism was 7–11% [9,10]. In our study, the irreplaceable mortality due to parasitism was lower compared to wheat; as a result, the mortality from parasitism would most likely have been largely replaced by other mortality causes if parasitism was not present. Further, the discrepancies in the rate of parasitism are associated with cultivar traits. For instance, Buteler et al. [9] found a greater rate of parasitism in *C*. *cinctus* in a susceptible hollow stem wheat cultivar compared to a resistant solid stem wheat cultivar. The cultivars included in our study had a wide range of resistance levels against *C*. *cinctus* and that could explain the varying rates of parasitism by cultivars. This is not because plant resistance directly acted on the parasitoid females, but rather because it reduced the number of larval hosts available to parasitoid females. Thus, cultivars with greater levels of plant defense killed neonates, which reduced the number of larval hosts available to parasitoid females, ultimately causing a lower rate of parasitism in those cultivars.

Further, foraging parasitoids generally exploit visual and olfactory cues as well as optimized innate memories to detect their hosts [67]. Additionally, odor cues present on parasitoid pupal cocoons are important when developing host-seeking behaviors [68]. Thus, parasitoid adults might not be able to fully use cues from host *C*. *cinctus* larvae in infested barley. This is because common use of barley as a host by *C*. *cinctus* is relatively new, when compared to wheat at our study locations [4] and thus these specialist idiobiont parasitoids, with *C*. *cinctus* as their only known host, might have a reduced probability of locating large larvae in barley stems.

We conducted our research at two disparate locations. These locations have different agroclimatic conditions (e.g. the duration of crop growth was 115 days near Amsterdam in 2016 and only 99 days near Big Sandy in 2017). Therefore, we observed relative differences in larval mortality, as well as the expression of host-plant performance related to larval mortality, within cultivars across our study sites. However, the overall pattern we observed across the cultivars remains clear, where we see that there is a major tradeoff between plant defense and cannibalism as causes of irreplaceable mortality (Table 2, Fig 5, S1–S4 Figs).

Finally, our overall results suggest that cannibalism and plant defense were the two major causes of mortality for pre-diapause larvae across study sites. Further, irreplaceable mortality due to these two factors has a significant impact that reduces the size of pre-diapause larval populations of *C*. *cinctus*, which may drive the population growth the following year. Both of these causes of mortality are mediated by host plant traits, either relative preference [26] or antibiosis (Fig 2). In the future, transcriptomic and metabolomic studies could reveal the biochemical pathways that cause a greater proportion of neonate mortality (antibiosis) and those that result in obligate cannibalism when attracting more ovipositing females [23,42]. Thus, cultivars with both of these traits are critical to reducing economic losses caused by this species.

## Supporting information

S1 Fig Correlation coefficients of mortality percentages for given causes of mortality in the presence of other causes of mortality, combined over site × years and cultivars. (TIF)

S2 Fig Correlation coefficients of irreplaceable mortality percentages of given causes of mortality combined over site × years and cultivars. (TIF)

S3 Fig Correlation of mortality percentage caused by plant defense and cannibalism in the presence of other causes of mortality by cultivar, combined over site × years. Each solid circle represents a replication of each site × years. R indicates the correlation coefficient and shaded area around the blue line indicates standard errors of the regression line for each cultivar.
(TIF)

S4 Fig Correlation between irreplaceable mortality percentages of cannibalism and plant defense by cultivar, combined over site × years. Each solid circle represents a replication of each site × years. R indicates the correlation coefficient and shaded area around the blue line indicates standard errors of regression line for each cultivar.
(TIF)

S1 Table Estimated total mortality in *C*. *cinctus* for different cause-specific combinations. (DOCX)

S2 Table Multiple decrement life tables of wheat stem sawfly, *Cephus cinctus*, in barley cultivars that were grown at Amsterdam 2016 and 2017, and Big Sandy 2017 in Montana. (DOCX)

## References

[1] Morrill WL, Kushnak GD. Wheat stem sawfly (Hymenoptera: Cephidae) adaptation to winter sheat. Environmental Entomology. 1996;25(5):1128–32.

[2] Lesieur V, Martin JF, Weaver DK, Hoelmer KA, Smith DR, Morrill WL, et al Phylogeography of the wheat stem sawfly, *Cephus cinctus* Norton (Hymenoptera: Cephidae): Implications for pest management. PLoS One. 2016(12):e0168370. 27959958 10.1371/journal.pone.0168370 PMC5154603

[3] Achhami BB, Reddy GVP, Sherman JD, Peterson RKD, Weaver DK. Effect of precipitation and temperature on larval survival of *Cephus cinctus* (Hymenoptera: Cephidae) in barley cultivars. Journal of Economic Entomology. 2020: 10.1093/jee/toaa097 32424403

[4] Varella AC, Talbert LE, Achhami BB, Blake NK, Hofland ML, Sherman JD, et al Characterization of resistance to *Cephus cinctus* (Hymenoptera: Cephidae) in barley germplasm. Journal of Economic Entomology. 2018;111(2):923–30. 29474649 10.1093/jee/toy025 PMC6019026

[5] Beres BL, Carcamo HA, Weaver DK, Dosdall LM, Evenden ML, Hill BD, et al Integrating the building blocks of agronomy and biocontrol into an IPM strategy for wheat stem sawfly. Prairie Soils and Crops. 4:54–65.

[6] Church NS. Moisture and diapause in the wheat stem sawfly, Ce*phus cinctus* Nort. (Hymenoptera: Cephidae). The Canadian Entomologist. 1955;87(2):85–97.

[7] Holmes N. Effects of moisture, gravity, and light on the behavior of larvae of the wheat stem sawfly, *Cephus cinctus* (Hymenoptera: Cephidae). The Canadian Entomologist. 1975;107(4):391–401.

[8] Carey JR. The multiple decrement life table—A unifying framework for cause-of-death analysis in ecology. Oecologia. 1989;78(1):131–7. 28311912 10.1007/BF00377208

[9] Buteler M, Peterson RK, Hofland ML, Weaver DK. A multiple decrement life table reveals that host plant resistance and parasitism are major causes of mortality for the wheat stem sawfly. Environmental Entomology. 2015;44(6):1571–80. 26314030 10.1093/ee/nvv128

[10] Peterson RKD, Buteler M, Weaver DK, Macedo TB, Sun ZT, Perez OG, et al Parasitism and the demography of wheat stem sawfly larvae, C*ephus cinctus*. BioControl. 2011;56(6):831–9.

[11] Runyon JB, Morrill WL, Weaver DK, Miller PR. Parasitism of the wheat stem sawfly (Hymenoptera: Cephidae) by *Bracon cephi* and *B*. *lissogaster* (Hymenoptera: Braconidae) in wheat fields bordering tilled and untilled fallow in Montana. Journal of Economic Entomology. 2002;95(6):1130–34. 12539822 10.1603/0022-0493-95.6.1130

[12] Holmes N. Population dynamics of the wheat stem sawfly, *Cephus cinctus* (Hymenoptera: Cephidae), in wheat. The Canadian Entomologist. 1982;114(9):775–88.

[13] Holmes N, Peterson L. Effects of variety and date of seeding spring wheats and location in the field on sex ratio of the wheat stem sawfly, *Cephus cinctus* Nort.(Hymenoptera: Cephidae). Canadian Journal of Zoology. 1963;41(7):1217–22.

[14] Weaver DK, Nansen C, Runyon JB, Sing SE, Morrill WL. Spatial distributions of *Cephus cinctus* Norton (Hymenoptera: Cephidae) and its braconid parasitoids in Montana wheat fields. Biological Control. 2005;34(1):1–11.

[15] USDA. U. S. Department of Agriculture, Crop production 2019 summary. National Agricultural Statistics Service 2020. Available from: https://www.nass.usda.gov/Publications/Todays_Reports/reports/cropan20.pdf.

[16] Adhikari S, Seipel T, Menalled FD, Weaver DK. Farming system and wheat cultivar affect infestation of, and parasitism on, *Cephus cinctus* in the Northern Great Plains. Pest Management Science. 2018;74(11):2480–7. 29582553 10.1002/ps.4925

[17] Beres BL, Cárcamo HA, Byers JR, Clarke FR, Ruan Y, Pozniak CJ, et al Host plant interactions between wheat germplasm source and wheat stem sawfly *Cephus cinctus* Norton (Hymenoptera: Cephidae). II. Other germplasm. Canadian Journal of Plant Science. 2013;93(6):1169–77.

[18] Talbert LE, Sherman JD, Hofland ML, Lanning SP, Blake NK, Grabbe R, et al Resistance to *Cephus cinctus* Norton, the wheat stem sawfly, in a recombinant inbred line population of wheat derived from two resistance sources. Plant Breeding. 2014;133(4):427–32.

[19] Bekkerman A, Weaver DK. Modeling joint dependence of managed ecosystems pests: the case of the wheat stem sawfly. Journal of Agricultural and Resource Economics. 2018;43(2):172–94.

[20] AMBA. American Malt Barley Association, Barley variety survey—2019. 2020. Avalilable from: https://ambainc.org/wp-content/uploads/2020/01/2019-US-VARIETY-MAPS.pdf. Revised January, 2020.

[21] Piesik D, Weaver DK, Runyon JB, Buteler M, Peck GE, Morrill WL. Behavioural responses of wheat stem sawflies to wheat volatiles. Agricultural and Forest Entomology. 2008;10(3):245–53.

[22] Buteler M, Weaver DK, Peterson RK. Oviposition behavior of the wheat stem sawfly when encountering plants infested with cryptic conspecifics. Environmental Entomology. 2009;38(6):1707–15. 20021767 10.1603/022.038.0624

[23] Buteler M, Weaver DK. Host selection by the wheat stem sawfly in winter wheat and the role of semiochemicals mediating oviposition preference. Entomologia Experimentalis et Applicata. 2012;143(2):138–47.

[24] Holmes ND. Food relations of the wheat stem sawfly, *Cephus cinctus* Nort. (Hymenoptera: Cephidae). The Canadian Entomologist. 1954;86(4):159–67.

[25] Varella AC, Weaver DK, Peterson RK, Sherman JD, Hofland ML, Blake NK, et al Host plant quantitative trait loci affect specific behavioral sequences in oviposition by a stem-mining insect. Theoretical Applied Genetics. 2017;130(1):187–97. 27709252 10.1007/s00122-016-2805-0

[26] Weaver DK, Buteler M, Hofland ML, Runyon JB, Nansen C, Talbert LE, et al Cultivar preferences of ovipositing wheat stem sawflies as influenced by the amount of volatile attractant. Journal of Economic Entomology. 2009;102(3):1009–17. 19610414 10.1603/029.102.0320

[27] Zadoks JC, Chang TT, Konzak CF. A decimal code for the growth stages of cereals. Weed Research. 1974;14(6):415–21.

[28] Wallace LE, McNeal FH. Stem sawflies of economic importance in grain crops in the United States. USDA Technical Bulletin No. 1350. p.50; 1966.

[29] Weiss MJ, Morrill WL. Wheat stem sawfly (Hymenoptera: Cephidae) revisited. American Entomologist. 1992;38(4):241–5.

[30] Morrill WL, Weaver DK, Irish NJ, Barr WF. *Phyllobaenus dubius* (Wolcott) (Coleoptera: Cleridae), a new record of a predator of the wheat stem sawfly (Hymenoptera: Cephidae). Journal of Kansas Entomological Society. 2001;74(3):181–3.

[31] Wenda-Piesik A, Sun Z, Grey WE, Weaver DK, Morrill WL. Mycoses of wheat stem sawfly (Hymenoptera: Cephidae) larvae by *Fusarium* spp. isolates. Environmental Entomology. 2009;38(2):387–94. 19389287 10.1603/022.038.0211

[32] Carey JR. Applied demography for biologist. 1st ed. New York: Oxford University Press; 1993.

[33] Davis RS, Peterson RKD, Higley LG. M-DEC: A spreadsheet program for producing multiple decrement life tables and estimating mortality dynamics for insects. Computers and Electronics in Agriculture. 2011;75(2):363–7.

[34] Peterson RKD, Davis RS, Higley LG, Fernandes OA. Mortality risk in insects. Environmental Entomology. 2009;38(1):2–10. 19791592 10.1603/022.038.0102

[35] Pinheiro J, D. Bates, S. DebRoy, D. Sarkar D, Team RC. nlme: linear and nonlinear mixed effects models, R package version 3.1–147. 2020.

[36] Hothorn T, Bretz F, Westfall P. Simultaneous inference in general parametric models. Biometrical Journal. 2008;50(3):346–63. 18481363 10.1002/bimj.200810425

[37] Hadley W, Seidel D. scales: Scale functions for visualization. Available from: https://scales.r-lib.org, https://github.com/r-lib/scales. 2019.

[38] Kassambara A. ggpubr: "ggplot2" based publication ready plots (Version 0.1. 7). 2017.

[39] Kassambara A. ggcorrplot: Visualization of a correlation matrix using 'ggplot2'. 0.1.3 ed2020.

[40] R Core Team. R: A language and environment for statistical computing. R Foundation for statistical computing, Vienna, Austria Available from: https://www.R-project.org/. 2019.

[41] Wickham H. ggplot2: Elegant graphics for data analysis. 3^rd^ ed New York: Springer- Verlag; 2016.

[42] Buteler M, Weaver DK, Bruckner PL, Carlson GR, Berg JE, Lamb PF. Using agronomic traits and semiochemical production in winter wheat cultivars to identify suitable trap crops for the wheat stem sawfly. The Canadian Entomologist. 2010;142(3):222–33.

[43] Ainslie CN. The western grass-stem sawfly: US Department of Agriculture. Bulletin no. 841. p.27; 1920.

[44] Farstad C. Wheat stem sawfly in flax. Scientific Agriculture. 1944;24(8):383–6.

[45] Holmes ND, Peterson LK. The influence of the host on oviposition by the wheat stem sawfly, *Cephus cinctus* Nort. (Hymenoptera: Cephidae). Canadian Journal of Plant Science. 1960;40(1):29–46.

[46] Kakimoto T, Fujisaki K, Miyatake T. Egg laying preference, larval dispersion, and cannibalism in *Helicoverpa armigera* (Lepidoptera: Noctuidae). Annals of the Entomological Society of America. 2003;96(6):793–8.

[47] Dickinson JL. Egg cannibalism by larvae and adults of the milkweed leaf beetle (*Labidomera clivicollis*, Coleoptera: Chrysomelidae). Ecological Entomology. 1992;17(3):209–18.

[48] Lopez-Ortega M, Williams T. Natural enemy defense, provisioning and oviposition site selection as maternal strategies to enhance offspring survival in a sub-social bug. PLoS One. 2018;13(4): e0195665. 29694361 10.1371/journal.pone.0195665 PMC5918792

[49] Sherratt TN, Ruff SE, Church SC. No evidence for kin discrimination in cannibalistic tree-hole mosquitoes (Diptera: Culicidae). Journal of Insect Behavior. 1999;12(1):123–32.

[50] Pfennig D, Ho S, Hoffman E. Pathogen transmission as a selective force against cannibalism. Animal Behaviour. 1998;55(5):1255–61. 9632508 10.1006/anbe.1997.9996

[51] Wetzel WC, Kharouba HM, Robinson M, Holyoak M, Karban R. Variability in plant nutrients reduces insect herbivore performance. Nature. 2016;539(7629):425–7. 27749815 10.1038/nature20140

[52] Richardson ML, Mitchell RF, Reagel PF, Hanks LM. Causes and consequences of cannibalism in noncarnivorous insects. Annual Review of Entomology. 2010;55:39–53. 10.1146/annurev-ento-112408-085314 19961322

[53] Snyder WE, Joseph SB, Preziosi R, Moore AJ. Nutritional benefits of cannibalism for the lady beetle *Harmonia axyridis* (Coleoptera: Coccinellidae) when prey quality is poor. Environmental Entomology. 2000;29(6):1173–9.

[54] Farstad CW, Platt AW. The reaction of barley varieties to wheat stem sawfly attack. Scientific Agriculture. 1946;26(5):216–24.

[55] Wang XG, Daane KM. Cannibalism of parasitoid‐attacked conspecifics in a non‐carnivorous caterpillar. Entomologia Experimentalis et Applicata. 2014;151(2):112–21.

[56] Buteler M, Weaver DK, Miller PR. Wheat stem sawfly-infested plants benefit from parasitism of the herbivorous larvae. Agricultural and Forest Entomology. 2008;10(4):347–54.

[57] Polis GA. The evolution and dynamics of intraspecific predation. Annual Review of Ecology and Systematics. 1981;12(1):225–51.

[58] Sherman JD, Weaver DK, Hofland ML, Sing SE, Buteler M, Lanning SP, et al Identification of novel QTL for sawfly resistance in wheat. Crop Science. 2010;50:73–86.

[59] Bernays EA, Oppenheim S, Chapman RF, Kwon H, Gould F. Taste sensitivity of insect herbivores to deterrents is greater in specialists than in generalists: A behavioral test of the hypothesis with two closely related caterpillars. Journal of Chemical Ecology. 2000;26(2):547–63.

[60] Larsson KAE, Saheed SA, Gradin T, Delp G, Karpinska B, Botha CEJ, et al Differential regulation of 3-aminomethylindole/N-methyl-3-aminomethylindole N-methyltransferase and gramine in barley by both biotic and abiotic stress conditions. Plant Physiology and Biochemistry. 2011;49(1):96–102. 21074448 10.1016/j.plaphy.2010.10.005

[61] Zúǹiga GE, Salgado MS, Corcuera LJ. Role of an indole alkaloid in the resistance of barley seedlings to aphids. Phytochemistry. 1985;24(5):945–7.

[62] Ryan CA. Protease inhibitors in plants: genes for improving defenses against insects and pathogens. Annual Review of Phytopathology. 1990;28:425–49.

[63] Santamaria ME, Cambra I, Martinez M, Pozancos C, González-Melendi P, Grbic V, et al Gene pyramiding of peptidase inhibitors enhances plant resistance to the spider mite *Tetranychus urticae*. PLoS One. 2012;7(8):e43011. 22900081 10.1371/journal.pone.0043011 PMC3416837

[64] Portman SL, Jaronski ST, Weaver DK, Reddy GV. Advancing biological control of the wheat stem sawfly: new strategies in a 100-yr struggle to manage a costly pest in the Northern Great Plains. Annals of the Entomological Society of America. 2018;111(3):85–91.

[65] Vidal S, Jaber LR. Entomopathogenic fungi as endophytes: plant-endophyte-herbivore interactions and prospects for use in biological control. Current Science. 2015;109(1):46–54.

[66] Carcamo HA, Beres BL, Larson TR, Klima CL, Wu XH. Effect of wheat cultivars and blends on the oviposition and larval mortality of *Cephus cinctus* (Hymenoptera: Cephidae) and parasitism by *Bracon cephi* (Hymenoptera: Braconidae). Environmental Entomology. 2016;45(2):397–403. 26802117 10.1093/ee/nvv231

[67] Turlings TC, Wäckers FL, Vet LE, Lewis WJ, Tumlinson JH. Learning of host-finding cues by hymenopterous parasitoids Insect Learning: Springer; 1993 pp. 51–78.

[68] Hérard F, Keller M, Lewis W, Tumlinson J. Beneficial arthropod behavior mediated by airborne semiochemicals. Journal of Chemical Ecology. 1988;14(7):1583–96. 24276431 10.1007/BF01012524

