# Supplementary figures and images for: Multiple decrement life tables of Cephus cinctus Norton (Hymenoptera: Cephidae) across a set of barley cultivars: The importance of plant defense versus cannibalism
Source: PLoS One. 2020 Sep 11;15(9):e0238527. doi: 10.1371/journal.pone.0238527 (PMC7485797; doi:10.1371/journal.pone.0238527)

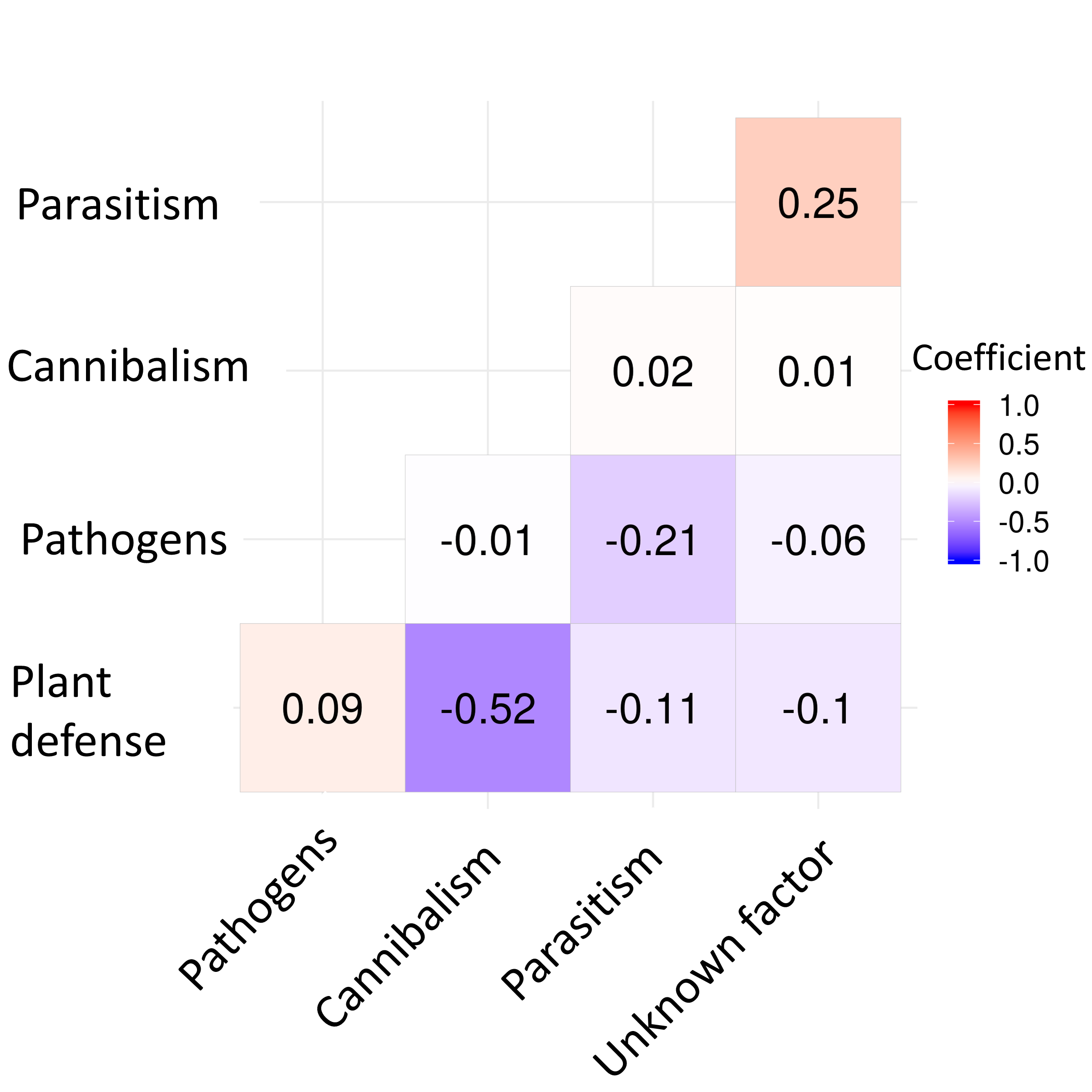

Supplement: S1 Fig — (TIF) [file pone.0238527.s001.tif]

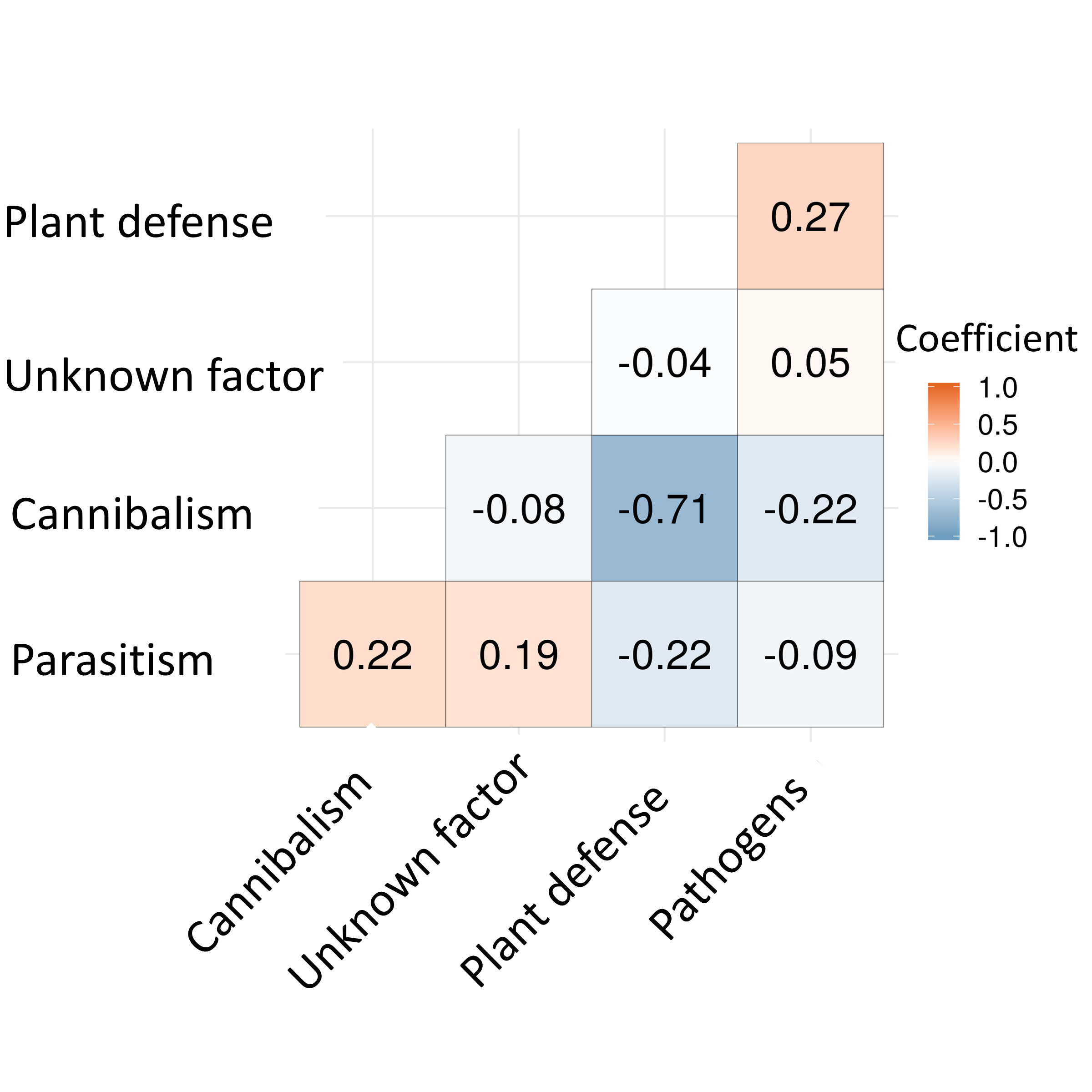

Supplement: S2 Fig — (TIF) [file pone.0238527.s002.tif]

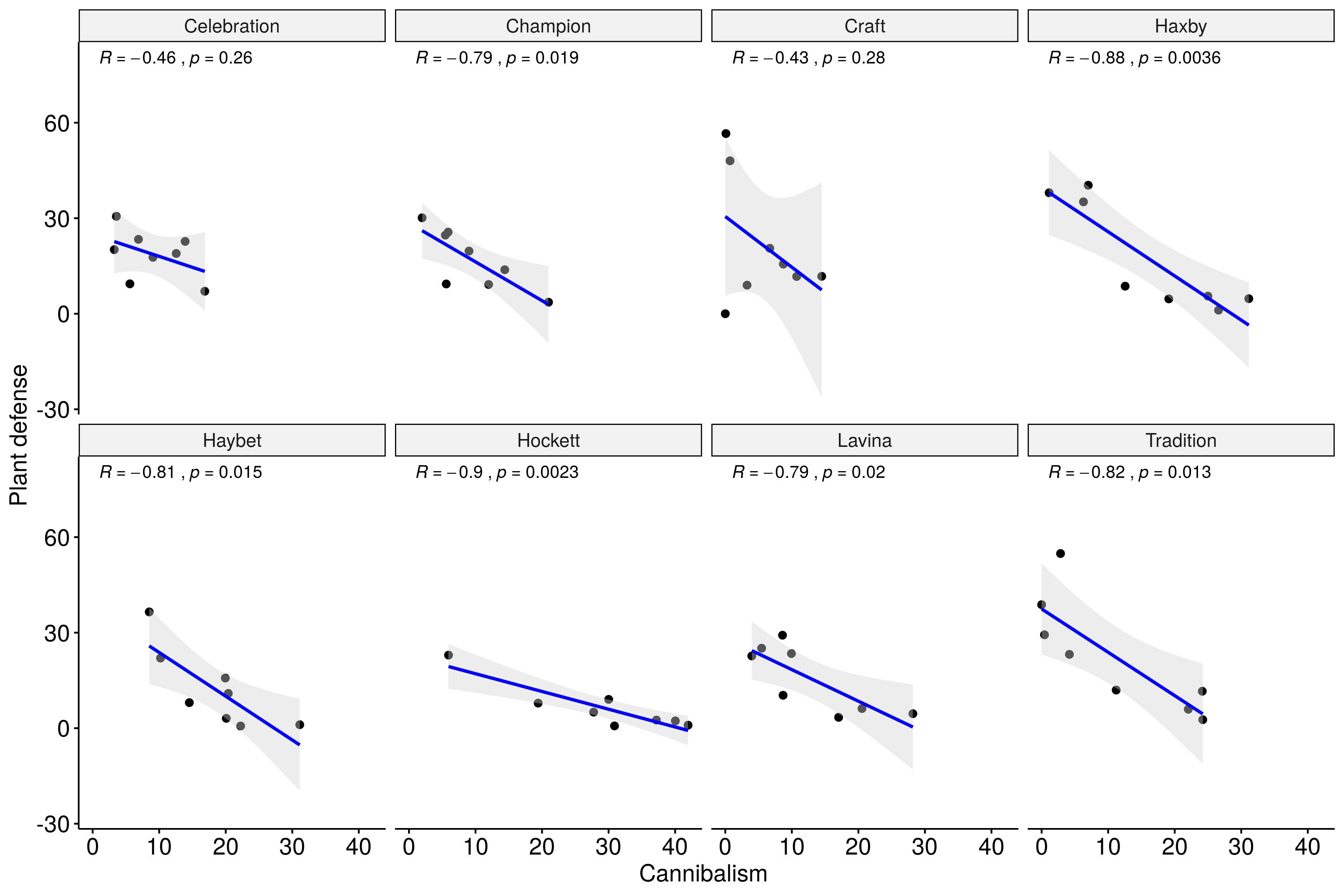

Supplement: S3 Fig — Each solid circle represents a replication of each site × years. R indicates the correlation coefficient and shaded area around the blue line indicates standard errors of the regression line for each cultivar. (TIF) [file pone.0238527.s003.tif]

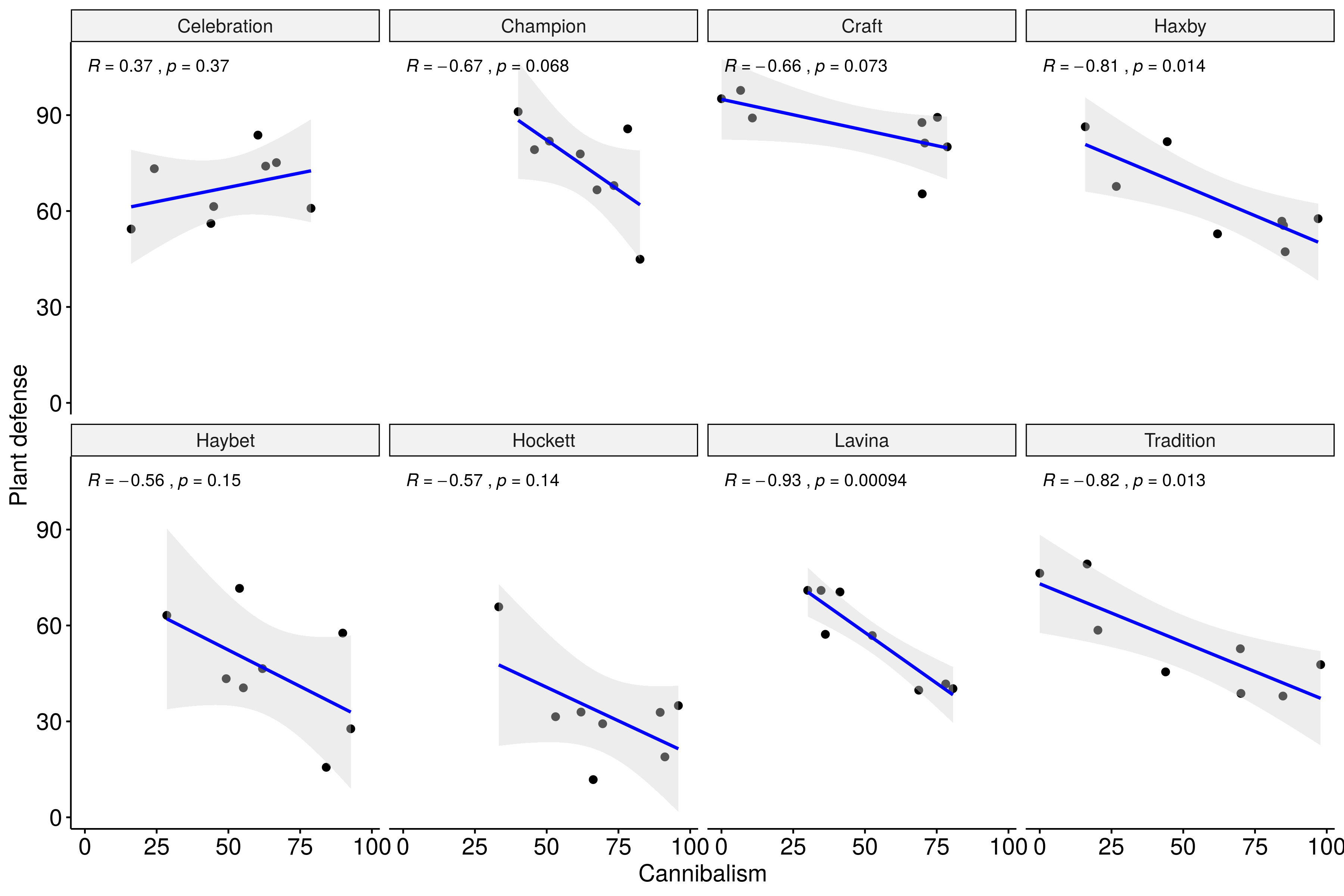

Supplement: S4 Fig — Each solid circle represents a replication of each site × years. R indicates the correlation coefficient and shaded area around the blue line indicates standard errors of regression line for each cultivar. (TIF) [file pone.0238527.s004.tif]
