# Supplementary material for: Multiple decrement life tables of Cephus cinctus Norton (Hymenoptera: Cephidae) across a set of barley cultivars: The importance of plant defense versus cannibalism
Source: PLoS One. 2020 Sep 11;15(9):e0238527. doi: 10.1371/journal.pone.0238527 (PMC7485797; doi:10.1371/journal.pone.0238527)
Supplement: S1 Table — (DOCX) [file pone.0238527.s005.docx]

**S1 Table.** Estimated total mortality in *C. cinctus* for different cause-specific combinations

| **Mortality causes** | **Number of causes** | **Estimated total mortality** |
| --- | --- | --- |
| Parasitism | 1 | 0.104 |
| Plant defense | 1 | 0.573 |
| Cannibalism | 1 | 0.717 |
| Unknown factors | 1 | 0.355 |
| Pathogens | 1 | 0.027 |
| Parasitism + Plant defense | 2 | 0.617 |
| Parasitism + Cannibalism | 2 | 0.746 |
| Parasitism + Unknown factors | 2 | 0.422 |
| Plant defense + Cannibalism | 2 | 0.879 |
| Plant defense + Unknown factors | 2 | 0.724 |
| Cannibalism + Unknown factors | 2 | 0.817 |
| Parasitism + Pathogens | 2 | 0.128 |
| Plant defense + Pathogens | 2 | 0.584 |
| Cannibalism + Pathogens | 2 | 0.724 |
| Unknown factors + Pathogens | 2 | 0.372 |
| Parasitism + Plant defense + Cannibalism | 3 | 0.892 |
| Parasitism + Plant defense + Unknown factors | 3 | 0.753 |
| Parasitism + Cannibalism + Unknown factors | 3 | 0.836 |
| Plant defense + Cannibalism + Unknown factors | 3 | 0.922 |
| Parasitism+ Plant defense + Pathogens | 3 | 0.627 |
| Parasitism+ Cannibalism + Pathogens | 3 | 0.753 |
| Parasitism+ Unknown factors + Pathogens | 3 | 0.438 |
| Plant defense + Cannibalism + Pathogens | 3 | 0.882 |
| Plant defense + Unknown factors + Pathogens | 3 | 0.732 |
| Cannibalism + Unknown factors + Pathogens | 3 | 0.822 |
| Parasitism + Plant defense + Cannibalism + Unknown factors | 4 | 0.930 |
| Parasitism + Plant defense + Cannibalism + Pathogens | 4 | 0.894 |
| Parasitism + Plant defense + Unknown factors + Pathogens | 4 | 0.760 |
| Parasitism + Cannibalism + Unknown factors + Pathogens | 4 | 0.841 |
| Plant defense + Cannibalism + Unknown factors + Pathogens | 4 | 0.924 |
| Parasitism + Plant defense + Cannibalism + Unknown factors + Pathogens | 5 | 0.932 |

Proportion of mortality estimated by one cause of mortality or by combination of more than one cause of mortality using M-DEC spread sheet (33).
