# Supplementary material for: Multiple decrement life tables of Cephus cinctus Norton (Hymenoptera: Cephidae) across a set of barley cultivars: The importance of plant defense versus cannibalism
Source: PLoS One. 2020 Sep 11;15(9):e0238527. doi: 10.1371/journal.pone.0238527 (PMC7485797; doi:10.1371/journal.pone.0238527)
Supplement: S2 Table — (DOCX) [file pone.0238527.s006.docx]

**S2 Table.** Multiple decrement life tables of wheat stem sawfly, *Cephus cinctus*, in barley cultivars that were grown at Amsterdam 2016 and 2017, and Big Sandy 2017 in Montana

| **Location** | **Cultivar** | **Category,**  **x** | **Proportion Dying,**  **aqx** | **Proportion of living,**  **alx** | **Proportion**  **of total**  **dying,**  **adx** | | **Parasitism,**  **aq1x** | **Plant defense, aq2x** | | **Cannibalism,**  **aq3x** | **Unknown,**  **aq4x** | | **Pathogens,**  **aq5x** |
| --- | --- | --- | --- | --- | --- | --- | --- | --- | --- | --- | --- | --- | --- |
| Amsterdam 2016 | Celebration | Egg | 0.24 | 1.00 | 0.24 | | 0.00 | 0.00 | | 0.24 | 0.00 | | 0.00 |
|  |  | Larva I | 0.51 | 0.76 | 0.39 | | 0.00 | 0.31 | | 0.05 | 0.03 | | 0.00 |
|  |  | Larva III | 0.49 | 0.37 | 0.18 | | 0.01 | 0.09 | | 0.00 | 0.07 | | 0.00 |
|  |  | Larva IV | 0.18 | 0.19 | 0.03 | | 0.00 | 0.00 | | 0.00 | 0.03 | | 0.00 |
|  |  | Larva V | 0.16 | 0.15 | 0.02 | | 0.00 | 0.00 | | 0.00 | 0.02 | | 0.00 |
|  |  | Adult | 0.00 | 0.13 | 0.13 | | 0.00 | 0.00 | | 0.00 | 0.00 | | 0.00 |
|  |  | Total |  |  |  | | 0.02 | 0.40 | | 0.30 | 0.15 | | 0.00 |
|  | Champion | Egg | 0.41 | 1.00 | 0.41 | | 0.00 | 0.00 | | 0.41 | 0.00 | | 0.00 |
|  |  | Larva I | 0.63 | 0.59 | 0.37 | | 0.00 | 0.29 | | 0.05 | 0.04 | | 0.00 |
|  |  | Larva III | 0.67 | 0.22 | 0.15 | | 0.00 | 0.11 | | 0.00 | 0.03 | | 0.00 |
|  |  | Larva IV | 0.14 | 0.07 | 0.01 | | 0.00 | 0.00 | | 0.00 | 0.01 | | 0.00 |
|  |  | Larva V | 0.04 | 0.06 | 0.00 | | 0.00 | 0.00 | | 0.00 | 0.00 | | 0.00 |
|  |  | Adult | 0.00 | 0.06 | 0.06 | | 0.00 | 0.00 | | 0.00 | 0.00 | | 0.00 |
|  |  | Total |  |  |  | | 0.00 | 0.40 | | 0.46 | 0.08 | | 0.00 |
|  | Craft | Egg | 0.06 | 1.00 | 0.06 | | 0.00 | 0.00 | | 0.06 | 0.00 | | 0.00 |
|  |  | Larva I | 0.78 | 0.94 | 0.74 | | 0.00 | 0.70 | | 0.01 | 0.03 | | 0.00 |
|  |  | Larva III | 0.79 | 0.21 | 0.16 | | 0.00 | 0.14 | | 0.00 | 0.03 | | 0.00 |
|  |  | Larva IV | 0.29 | 0.04 | 0.01 | | 0.01 | 0.00 | | 0.00 | 0.00 | | 0.00 |
|  |  | Larva V | 0.20 | 0.03 | 0.01 | | 0.01 | 0.00 | | 0.00 | 0.00 | | 0.00 |
|  |  | Adult | 0.00 | 0.02 | 0.02 | | 0.00 | 0.00 | | 0.00 | 0.00 | | 0.00 |
|  |  | Total |  |  |  | | 0.02 | 0.84 | | 0.06 | 0.06 | | 0.00 |
|  | Haxby | Egg | 0.27 | 1.00 | 0.27 | | 0.00 | 0.00 | | 0.27 | 0.00 | | 0.00 |
|  |  | Larva I | 0.59 | 0.73 | 0.43 | | 0.00 | 0.34 | | 0.06 | 0.03 | | 0.00 |
|  |  | Larva III | 0.60 | 0.30 | 0.18 | | 0.00 | 0.13 | | 0.01 | 0.04 | | 0.00 |
|  |  | Larva IV | 0.03 | 0.12 | 0.00 | | 0.00 | 0.00 | | 0.00 | 0.00 | | 0.00 |
|  |  | Larva V | 0.00 | 0.12 | 0.00 | | 0.00 | 0.00 | | 0.00 | 0.00 | | 0.00 |
|  |  | Adult | 0.00 | 0.12 | 0.12 | | 0.00 | 0.00 | | 0.00 | 0.00 | | 0.00 |
|  |  | Total |  |  |  | | 0.00 | 0.47 | | 0.34 | 0.07 | | 0.00 |
|  | Haybet | Egg | 0.33 | 1.00 | 0.33 | | 0.00 | 0.00 | | 0.33 | 0.00 | | 0.00 |
|  |  | Larva I | 0.47 | 0.67 | 0.32 | | 0.00 | 0.18 | | 0.11 | 0.03 | | 0.00 |
|  |  | Larva III | 0.50 | 0.36 | 0.18 | | 0.01 | 0.12 | | 0.01 | 0.04 | | 0.00 |
|  |  | Larva IV | 0.09 | 0.18 | 0.02 | | 0.02 | 0.00 | | 0.00 | 0.00 | | 0.00 |
|  |  | Larva V | 0.00 | 0.16 | 0.00 | | 0.00 | 0.00 | | 0.00 | 0.00 | | 0.00 |
|  |  | Adult | 0.00 | 0.16 | 0.16 | | 0.00 | 0.00 | | 0.00 | 0.00 | | 0.00 |
|  |  | Total |  |  |  | | 0.02 | 0.30 | | 0.44 | 0.07 | | 0.00 |
|  | Hockett | Egg | 0.45 | 1.00 | 0.45 | | 0.00 | 0.00 | | 0.45 | 0.00 | | 0.00 |
|  |  | Larva I | 0.47 | 0.55 | 0.26 | | 0.00 | 0.12 | | 0.11 | 0.02 | | 0.00 |
|  |  | Larva III | 0.33 | 0.29 | 0.10 | | 0.00 | 0.05 | | 0.01 | 0.04 | | 0.00 |
|  |  | Larva IV | 0.12 | 0.20 | 0.02 | | 0.00 | 0.00 | | 0.00 | 0.02 | | 0.00 |
|  |  | Larva V | 0.12 | 0.17 | 0.02 | | 0.01 | 0.00 | | 0.00 | 0.01 | | 0.00 |
|  |  | Adult | 0.00 | 0.15 | 0.15 | | 0.00 | 0.00 | | 0.00 | 0.00 | | 0.00 |
|  |  | Total |  |  |  | | 0.02 | 0.17 | | 0.57 | 0.09 | | 0.00 |
|  | Lavina | Egg | 0.27 | 1.00 | 0.27 | | 0.00 | 0.00 | | 0.27 | 0.00 | | 0.00 |
|  |  | Larva I | 0.54 | 0.73 | 0.40 | | 0.00 | 0.30 | | 0.08 | 0.03 | | 0.00 |
|  |  | Larva III | 0.60 | 0.34 | 0.20 | | 0.01 | 0.13 | | 0.01 | 0.06 | | 0.00 |
|  |  | Larva IV | 0.20 | 0.13 | 0.03 | | 0.00 | 0.00 | | 0.00 | 0.03 | | 0.00 |
|  |  | Larva V | 0.50 | 0.11 | 0.05 | | 0.05 | 0.00 | | 0.00 | 0.00 | | 0.00 |
|  |  | Adult | 0.00 | 0.05 | 0.05 | | 0.00 | 0.00 | | 0.00 | 0.00 | | 0.00 |
|  |  | Total |  |  |  | | 0.06 | 0.43 | | 0.35 | 0.11 | | 0.00 |
|  | Tradition | Egg | 0.05 | 1.00 | 0.05 | | 0.00 | 0.00 | | 0.05 | 0.00 | | 0.00 |
|  |  | Larva I | 0.49 | 0.95 | 0.46 | | 0.00 | 0.35 | | 0.09 | 0.03 | | 0.00 |
|  |  | Larva III | 0.61 | 0.48 | 0.30 | | 0.00 | 0.20 | | 0.01 | 0.09 | | 0.00 |
|  |  | Larva IV | 0.08 | 0.19 | 0.01 | | 0.00 | 0.00 | | 0.00 | 0.01 | | 0.00 |
|  |  | Larva V | 0.02 | 0.17 | 0.00 | | 0.00 | 0.00 | | 0.00 | 0.00 | | 0.00 |
|  |  | Adult | 0.00 | 0.17 | 0.17 | | 0.00 | 0.00 | | 0.00 | 0.00 | | 0.00 |
|  |  | Total |  |  |  | | 0.00 | 0.55 | | 0.14 | 0.13 | | 0.00 |
| Amsterdam 2017 | Celebration | Egg | 0.63 | 1.00 | | 0.63 | 0.00 | | 0.00 | 0.63 | | 0.00 | 0.00 |
|  |  | Larva I | 0.49 | 0.37 | | 0.18 | 0.00 | | 0.13 | 0.05 | | 0.00 | 0.00 |
|  |  | Larva III | 0.50 | 0.19 | | 0.09 | 0.01 | | 0.06 | 0.01 | | 0.02 | 0.00 |
|  |  | Larva IV | 0.18 | 0.10 | | 0.02 | 0.01 | | 0.00 | 0.00 | | 0.01 | 0.00 |
|  |  | Larva V | 0.17 | 0.08 | | 0.01 | 0.01 | | 0.00 | 0.00 | | 0.01 | 0.00 |
|  |  | Adult | 0.00 | 0.06 | | 0.06 | 0.00 | | 0.00 | 0.00 | | 0.00 | 0.00 |
|  |  | Total |  |  | |  | 0.02 | | 0.19 | 0.68 | | 0.04 | 0.00 |
|  | Champion | Egg | 0.69 | 1.00 | | 0.69 | 0.00 | | 0.00 | 0.69 | | 0.00 | 0.00 |
|  |  | Larva I | 0.82 | 0.31 | | 0.25 | 0.00 | | 0.16 | 0.09 | | 0.00 | 0.00 |
|  |  | Larva III | 0.56 | 0.06 | | 0.03 | 0.01 | | 0.02 | 0.00 | | 0.01 | 0.00 |
|  |  | Larva IV | 0.13 | 0.02 | | 0.00 | 0.00 | | 0.00 | 0.00 | | 0.00 | 0.00 |
|  |  | Larva V | 0.26 | 0.02 | | 0.01 | 0.00 | | 0.00 | 0.00 | | 0.00 | 0.00 |
|  |  | Adult | 0.00 | 0.02 | | 0.02 | 0.00 | | 0.00 | 0.00 | | 0.00 | 0.00 |
|  |  | Total |  |  | |  | 0.01 | | 0.17 | 0.79 | | 0.02 | 0.00 |
|  | Craft | Egg | 0.64 | 1.00 | | 0.64 | 0.00 | | 0.00 | 0.64 | | 0.00 | 0.00 |
|  |  | Larva I | 0.57 | 0.36 | | 0.20 | 0.00 | | 0.15 | 0.05 | | 0.00 | 0.00 |
|  |  | Larva III | 0.62 | 0.15 | | 0.09 | 0.01 | | 0.07 | 0.00 | | 0.01 | 0.00 |
|  |  | Larva IV | 0.17 | 0.06 | | 0.01 | 0.00 | | 0.00 | 0.00 | | 0.01 | 0.00 |
|  |  | Larva V | 0.10 | 0.05 | | 0.00 | 0.00 | | 0.00 | 0.00 | | 0.00 | 0.00 |
|  |  | Adult | 0.00 | 0.04 | | 0.04 | 0.00 | | 0.00 | 0.00 | | 0.00 | 0.00 |
|  |  | Total |  |  | |  | 0.01 | | 0.22 | 0.70 | | 0.03 | 0.00 |
|  | Haxby | Egg | 0.77 | 1.00 | | 0.77 | 0.00 | | 0.00 | 0.77 | | 0.00 | 0.00 |
|  |  | Larva I | 0.50 | 0.23 | | 0.11 | 0.00 | | 0.06 | 0.05 | | 0.00 | 0.00 |
|  |  | Larva III | 0.54 | 0.11 | | 0.06 | 0.01 | | 0.03 | 0.01 | | 0.01 | 0.00 |
|  |  | Larva IV | 0.10 | 0.05 | | 0.01 | 0.00 | | 0.00 | 0.00 | | 0.00 | 0.00 |
|  |  | Larva V | 0.19 | 0.05 | | 0.01 | 0.00 | | 0.00 | 0.00 | | 0.01 | 0.00 |
|  |  | Adult | 0.00 | 0.04 | | 0.04 | 0.00 | | 0.00 | 0.00 | | 0.00 | 0.00 |
|  |  | Total |  |  | |  | 0.01 | | 0.09 | 0.83 | | 0.02 | 0.00 |
|  | Haybet | Egg | 0.83 | 1.00 | | 0.83 | 0.00 | | 0.00 | 0.83 | | 0.00 | 0.00 |
|  |  | Larva I | 0.42 | 0.17 | | 0.07 | 0.00 | | 0.02 | 0.05 | | 0.00 | 0.00 |
|  |  | Larva III | 0.53 | 0.10 | | 0.05 | 0.01 | | 0.01 | 0.01 | | 0.02 | 0.00 |
|  |  | Larva IV | 0.20 | 0.05 | | 0.01 | 0.00 | | 0.00 | 0.00 | | 0.01 | 0.00 |
|  |  | Larva V | 0.23 | 0.04 | | 0.01 | 0.00 | | 0.00 | 0.00 | | 0.01 | 0.00 |
|  |  | Adult | 0.00 | 0.03 | | 0.03 | 0.00 | | 0.00 | 0.00 | | 0.00 | 0.00 |
|  |  | Total |  |  | |  | 0.02 | | 0.03 | 0.88 | | 0.04 | 0.00 |
|  | Hockett | Egg | 0.81 | 1.00 | | 0.81 | 0.00 | | 0.00 | 0.81 | | 0.00 | 0.00 |
|  |  | Larva I | 0.41 | 0.19 | | 0.08 | 0.00 | | 0.02 | 0.06 | | 0.00 | 0.00 |
|  |  | Larva III | 0.41 | 0.11 | | 0.05 | 0.01 | | 0.01 | 0.01 | | 0.01 | 0.00 |
|  |  | Larva IV | 0.08 | 0.07 | | 0.01 | 0.00 | | 0.00 | 0.00 | | 0.01 | 0.00 |
|  |  | Larva V | 0.11 | 0.06 | | 0.01 | 0.00 | | 0.00 | 0.00 | | 0.00 | 0.00 |
|  |  | Adult | 0.00 | 0.05 | | 0.05 | 0.00 | | 0.00 | 0.00 | | 0.00 | 0.00 |
|  |  | Total |  |  | |  | 0.01 | | 0.02 | 0.88 | | 0.02 | 0.00 |
|  | Lavina | Egg | 0.69 | 1.00 | | 0.69 | 0.00 | | 0.00 | 0.69 | | 0.00 | 0.00 |
|  |  | Larva I | 0.45 | 0.31 | | 0.14 | 0.00 | | 0.06 | 0.07 | | 0.00 | 0.00 |
|  |  | Larva III | 0.51 | 0.17 | | 0.09 | 0.03 | | 0.03 | 0.01 | | 0.02 | 0.00 |
|  |  | Larva IV | 0.26 | 0.08 | | 0.02 | 0.01 | | 0.00 | 0.00 | | 0.01 | 0.00 |
|  |  | Larva V | 0.16 | 0.06 | | 0.01 | 0.01 | | 0.00 | 0.00 | | 0.00 | 0.00 |
|  |  | Adult | 0.00 | 0.05 | | 0.05 | 0.00 | | 0.00 | 0.00 | | 0.00 | 0.00 |
|  |  | Total |  |  | |  | 0.04 | | 0.09 | 0.78 | | 0.04 | 0.00 |
|  | Tradition | Egg | 0.30 | 1.00 | | 0.30 | 0.00 | | 0.00 | 0.30 | | 0.00 | 0.00 |
|  |  | Larva I | 0.45 | 0.70 | | 0.32 | 0.00 | | 0.18 | 0.13 | | 0.00 | 0.00 |
|  |  | Larva III | 0.47 | 0.38 | | 0.18 | 0.03 | | 0.07 | 0.02 | | 0.06 | 0.00 |
|  |  | Larva IV | 0.13 | 0.20 | | 0.03 | 0.00 | | 0.00 | 0.00 | | 0.02 | 0.00 |
|  |  | Larva V | 0.13 | 0.17 | | 0.02 | 0.00 | | 0.00 | 0.00 | | 0.02 | 0.01 |
|  |  | Adult | 0.00 | 0.15 | | 0.15 | 0.00 | | 0.00 | 0.00 | | 0.00 | 0.00 |
|  |  | Total |  |  | |  | 0.04 | | 0.25 | 0.46 | | 0.10 | 0.01 |
| Big Sandy 2017 | Celebration | Egg | 0.55 | 1.00 | | 0.55 | 0.00 | | 0.00 | 0.55 | 0.00 | | 0.00 |
|  |  | Larva I | 0.45 | 0.45 | | 0.20 | 0.00 | | 0.12 | 0.06 | 0.02 | | 0.00 |
|  |  | Larva III | 0.73 | 0.24 | | 0.18 | 0.01 | | 0.14 | 0.01 | 0.03 | | 0.00 |
|  |  | Larva IV | 0.19 | 0.07 | | 0.01 | 0.00 | | 0.00 | 0.00 | 0.01 | | 0.00 |
|  |  | Larva V | 0.10 | 0.05 | | 0.01 | 0.00 | | 0.00 | 0.00 | 0.00 | | 0.00 |
|  |  | Adult | 0.00 | 0.05 | | 0.05 | 0.00 | | 0.00 | 0.00 | 0.00 | | 0.00 |
|  |  | Total |  |  | |  | 0.01 | | 0.26 | 0.62 | 0.06 | | 0.00 |
|  | Champion | Egg | 0.60 | 1.00 | | 0.60 | 0.00 | | 0.00 | 0.60 | 0.00 | | 0.00 |
|  |  | Larva I | 0.53 | 0.40 | | 0.21 | 0.00 | | 0.13 | 0.07 | 0.01 | | 0.00 |
|  |  | Larva III | 0.71 | 0.19 | | 0.13 | 0.00 | | 0.11 | 0.00 | 0.02 | | 0.00 |
|  |  | Larva IV | 0.17 | 0.06 | | 0.01 | 0.00 | | 0.00 | 0.00 | 0.01 | | 0.00 |
|  |  | Larva V | 0.14 | 0.05 | | 0.01 | 0.00 | | 0.00 | 0.00 | 0.00 | | 0.00 |
|  |  | Adult | 0.00 | 0.04 | | 0.04 | 0.00 | | 0.00 | 0.00 | 0.00 | | 0.00 |
|  |  | Total |  |  | |  | 0.00 | | 0.24 | 0.68 | 0.04 | | 0.00 |
|  | Craft | Egg | 0.70 | 1.00 | | 0.70 | 0.00 | | 0.00 | 0.70 | 0.00 | | 0.00 |
|  |  | Larva I | 0.47 | 0.30 | | 0.14 | 0.00 | | 0.10 | 0.04 | 0.00 | | 0.00 |
|  |  | Larva III | 0.81 | 0.16 | | 0.13 | 0.00 | | 0.11 | 0.00 | 0.01 | | 0.00 |
|  |  | Larva IV | 0.19 | 0.03 | | 0.01 | 0.00 | | 0.00 | 0.00 | 0.01 | | 0.00 |
|  |  | Larva V | 0.24 | 0.02 | | 0.01 | 0.00 | | 0.00 | 0.00 | 0.00 | | 0.00 |
|  |  | Adult | 0.00 | 0.02 | | 0.02 | 0.00 | | 0.00 | 0.00 | 0.00 | | 0.00 |
|  |  | Total |  |  | |  | 0.00 | | 0.21 | 0.74 | 0.03 | | 0.00 |
|  | Haxby | Egg | 0.70 | 1.00 | | 0.70 | 0.00 | | 0.00 | 0.70 | 0.00 | | 0.00 |
|  |  | Larva I | 0.44 | 0.30 | | 0.13 | 0.00 | | 0.05 | 0.08 | 0.01 | | 0.00 |
|  |  | Larva III | 0.59 | 0.17 | | 0.10 | 0.01 | | 0.06 | 0.00 | 0.03 | | 0.00 |
|  |  | Larva IV | 0.11 | 0.07 | | 0.01 | 0.00 | | 0.00 | 0.00 | 0.01 | | 0.00 |
|  |  | Larva V | 0.10 | 0.06 | | 0.01 | 0.00 | | 0.00 | 0.00 | 0.00 | | 0.00 |
|  |  | Adult | 0.00 | 0.06 | | 0.06 | 0.00 | | 0.00 | 0.00 | 0.00 | | 0.00 |
|  |  | Total |  |  | |  | 0.01 | | 0.11 | 0.78 | 0.05 | | 0.00 |
|  | Haybet | Egg | 0.52 | 1.00 | | 0.52 | 0.00 | | 0.00 | 0.52 | 0.00 | | 0.00 |
|  |  | Larva I | 0.38 | 0.48 | | 0.18 | 0.00 | | 0.07 | 0.11 | 0.00 | | 0.00 |
|  |  | Larva III | 0.57 | 0.29 | | 0.17 | 0.01 | | 0.10 | 0.01 | 0.05 | | 0.00 |
|  |  | Larva IV | 0.22 | 0.13 | | 0.03 | 0.00 | | 0.00 | 0.00 | 0.03 | | 0.00 |
|  |  | Larva V | 0.14 | 0.10 | | 0.01 | 0.00 | | 0.00 | 0.00 | 0.01 | | 0.00 |
|  |  | Adult | 0.00 | 0.08 | | 0.08 | 0.00 | | 0.00 | 0.00 | 0.00 | | 0.00 |
|  |  | Total |  |  | |  | 0.01 | | 0.16 | 0.65 | 0.09 | | 0.00 |
|  | Hockett | Egg | 0.73 | 1.00 | | 0.73 | 0.00 | | 0.00 | 0.73 | 0.00 | | 0.00 |
|  |  | Larva I | 0.42 | 0.27 | | 0.12 | 0.00 | | 0.04 | 0.08 | 0.00 | | 0.00 |
|  |  | Larva III | 0.33 | 0.16 | | 0.05 | 0.00 | | 0.03 | 0.01 | 0.01 | | 0.00 |
|  |  | Larva IV | 0.18 | 0.11 | | 0.02 | 0.00 | | 0.00 | 0.00 | 0.02 | | 0.00 |
|  |  | Larva V | 0.09 | 0.09 | | 0.01 | 0.00 | | 0.00 | 0.00 | 0.01 | | 0.00 |
|  |  | Adult | 0.00 | 0.08 | | 0.08 | 0.00 | | 0.00 | 0.00 | 0.00 | | 0.00 |
|  |  | Total |  |  | |  | 0.00 | | 0.07 | 0.82 | 0.03 | | 0.00 |
|  | Lavina | Egg | 0.41 | 1.00 | | 0.41 | 0.00 | | 0.00 | 0.41 | 0.00 | | 0.00 |
|  |  | Larva I | 0.37 | 0.59 | | 0.22 | 0.00 | | 0.08 | 0.13 | 0.00 | | 0.00 |
|  |  | Larva III | 0.56 | 0.38 | | 0.21 | 0.01 | | 0.13 | 0.01 | 0.06 | | 0.00 |
|  |  | Larva IV | 0.25 | 0.17 | | 0.04 | 0.00 | | 0.00 | 0.00 | 0.04 | | 0.00 |
|  |  | Larva V | 0.13 | 0.12 | | 0.02 | 0.00 | | 0.00 | 0.00 | 0.01 | | 0.00 |
|  |  | Adult | 0.00 | 0.11 | | 0.11 | 0.00 | | 0.00 | 0.00 | 0.00 | | 0.00 |
|  |  | Total |  |  | |  | 0.01 | | 0.22 | 0.55 | 0.11 | | 0.00 |
|  | Tradition | Egg | 0.57 | 1.00 | | 0.57 | 0.00 | | 0.00 | 0.57 | 0.00 | | 0.00 |
|  |  | Larva I | 0.40 | 0.43 | | 0.17 | 0.00 | | 0.07 | 0.09 | 0.01 | | 0.00 |
|  |  | Larva III | 0.50 | 0.26 | | 0.13 | 0.01 | | 0.07 | 0.01 | 0.03 | | 0.00 |
|  |  | Larva IV | 0.13 | 0.13 | | 0.02 | 0.00 | | 0.00 | 0.00 | 0.02 | | 0.00 |
|  |  | Larva V | 0.21 | 0.11 | | 0.02 | 0.00 | | 0.00 | 0.00 | 0.02 | | 0.01 |
|  |  | Adult | 0.00 | 0.09 | | 0.09 | 0.00 | | 0.00 | 0.00 | 0.00 | | 0.00 |
|  |  | Total |  |  | |  | 0.01 | | 0.14 | 0.67 | 0.08 | | 0.01 |

Larva I: pre-parasitism period; Larva III: parasitism period; Larva IV: overwintered larva (pre-flight period; post-parasitism); Larva V: overwintered larva (Post-flight period; post-parasitism).

aq_x_ = proportion of death caused by all mortality causes in stage x given that the individual is alive at the beginning of stage x; al_x_ = proportion of survivors at stage x out of the original cohort of all individuals; ad_x_ = fraction of deaths in stage x from all mortality causes; aq_ix_ = proportion of deaths from cause i in stage x in the presence of all other mortality causes, given that the individual is live at the beginning of state x.
